# Empirical patterns of environmental variation favor adaptive transgenerational plasticity

**DOI:** 10.1002/ece3.6022

**Published:** 2020-01-29

**Authors:** Jack M. Colicchio, Jacob Herman

**Affiliations:** ^1^ Department of Plant and Microbial Biology University of California Berkeley Berkeley CA USA; ^2^ Department of Organismic and Evolutionary Biology Harvard University Cambridge MA USA

**Keywords:** epigenetics, local adaptation, transgenerational plasticity

## Abstract

Effects of parental environment on offspring traits have been well known for decades. Interest in this transgenerational form of phenotypic plasticity has recently surged due to advances in our understanding of its mechanistic basis. Theoretical research has simultaneously advanced by predicting the environmental conditions that should favor the adaptive evolution of transgenerational plasticity. Yet whether such conditions actually exist in nature remains largely unexplored. Here, using long‐term climate data, we modeled optimal levels of transgenerational plasticity for an organism with a one‐year life cycle at a spatial resolution of 4 km^2^ across the continental United States. Both annual temperature and precipitation levels were often autocorrelated, but the strength and direction of these autocorrelations varied considerably even among nearby sites. When present, such environmental autocorrelations render offspring environments statistically predictable based on the parental environment, a key condition for the adaptive evolution of transgenerational plasticity. Results of our optimality models were consistent with this prediction: High levels of transgenerational plasticity were favored at sites with strong environmental autocorrelations, and little‐to‐no transgenerational plasticity was favored at sites with weak or nonexistent autocorrelations. These results are among the first to show that natural patterns of environmental variation favor the evolution of adaptive transgenerational plasticity. Furthermore, these findings suggest that transgenerational plasticity is likely variable in nature, depending on site‐specific patterns of environmental variation.

## Graphical abstract

Along with variation across the landscape for mean environmental conditions, here we report the presence of substantial spatial variation for the presence of temporal autocorrelations for temperature and precipitation. This variation in the autocorrelation of environments between years is likely to impact the types and strengths of transgenerational plasticity that evolve across the globe.

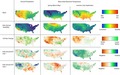

## 1 INTRODUCTION

Natural selection can produce adaptation only if the selective environment is reliably encountered over generations, or in other words, if selective environments are statistically predictable. Early models of evolution envisioned fitness landscapes that were static, such that populations adapt over the course of generations to one or another environment (Fisher, 1930). While this form of adaptation optimizes phenotypes in homogenous environments, the more realistic scenario of environmental heterogeneity in both space and time limits the adaptive value of such constitutive genetic expression (Sultan, 2015). In variable environments, the capacity to modify phenotypes in response to predictive environmental cues allows organisms to match their traits to the specific patch of habitat in which they find themselves, a phenomenon termed adaptive within‐generation plasticity (Ghalambor, McKay, Carroll, & Reznick, 2007; Nicotra et al., 2010). Investigating the predictability of environmental cues in nature is therefore a major research goal in ecology and evolution.

Over the last three decades, it has become clear that effects of parental environments on offspring phenotypes (i.e., *transgenerational plasticity*) are widespread and diverse (reviewed by Bonduriansky & Day, 2009; Conrath, Beckers, Langenbach, & Jaskiewicz, 2015; Holeski, Jander, & Agrawal, 2012; Mousseau & Fox, 1998; Sultan, 2015; Uller, 2008). For instance, when *Mimulus guttatus* plants experience herbivory, their offspring increase production of defensive leaf trichomes (Colicchio, 2017; Colicchio, Miura, Kelly, Ito, & Hileman, 2015; Holeski, 2007). Similarly, when the aquatic crustacean *Daphnia cucullata* senses predator cues, it produces offspring with a defensive “helmet” that protects against predation by midge larvae and cladocerans (Agrawal, Laforsch, & Tollrian, 1999). Such inherited environmental effects can be transmitted from parent to offspring (and to additional generations in some cases) by diverse and nonmutually exclusive mechanisms, including (a) heritable epigenetic modifications (i.e., DNA methylation marks, histone modifications, and small RNAs) and (b) the allocation of nutritive resources, hormones, mRNAs, and regulatory proteins to seeds or eggs (Herman & Sultan, 2011; Jablonka, 2013). As more research has focused on transgenerational plasticity, it has become clear that these effects are highly variable (Colicchio, 2017; Groot et al.., 2017; Herman & Sultan, 2016) and nearly absent in some cases (Ganguly, Crisp, Eichten, & Pogson, 2017). Empirical investigations in diverse plant and animal systems have confirmed that transgenerational environmental effects can be adaptive when parent and progeny environments match (i.e., under positive intergenerational environmental autocorrelations; see, e.g., Bilichak, Ilnystkyy, Hollunder, & Kovalchuk, 2012; Dantzer et al., 2013; Herman, Sultan, Horgan‐Kobelski, & Riggs, 2012; Lopez Sanchez, Stassen, Furci, Smith, & Ton, 2016; Rasmann et al., 2012; Slaughter et al., 2012; Verhoeven & van Gurp, 2012; Walsh et al., 2016; Wibowo et al., 2016). Further, Dey, Proulx, and Teotónio (2016), Graham, Smith, and Simons (2014), and Sikkink, Ituarte, Reynolds, Cresko, and Phillips (2014) have demonstrated that transgenerational plasticity can evolve in experimental settings.

These results motivated evolutionary research probing the theoretical scenarios in which transgenerational plasticity is expected to evolve adaptively. A central insight is that natural selection should favor specific forms of plasticity depending on the precise patterns of environmental variation experienced by a population (Shea, Pen, & Uller, 2011; Sultan & Spencer, 2002). Existing theory on the evolution of both within‐generation (Tufto, 2015; reviewed by Scheiner, 1993; Schlichting & Pigliucci, 1998) and transgenerational plasticity (Kuijper, Johnstone, & Townley, 2014; Lachmann & Jablonka, 1996; Leimar & McNamara, 2015; Prizak, Ezard, & Hoyle, 2014; Räsänen & Kruuk, 2007) has demonstrated that plasticity can evolve when three environmental conditions are met: The environment is correlated across time, there is little‐to‐no cost of responding to environmental cues, and there is genetic variation in reaction norm slope.

Transgenerational plasticity in particular is likely to evolve when parental and offspring conditions are (either positively or negatively) correlated (Proulx & Teotonio, 2017), with the magnitude of the correlation being the primary factor determining the optimal level of transgenerational plasticity. Recent models have shed light into the evolution of transgenerational plasticity in patchy environments (Leimar & McNamara, 2015), explicitly testing the conditions that favor deterministic versus. randomizing maternal effects (Proulx & Teotonio, 2017), studying how migration and population structure impact the evolution of transgenerational plasticity (Greenspoon & Spencer, 2018), determining the optimal levels of epigenetic resetting between generations (Uller, English, & Pen, 2015), and calculating the interaction between the evolution of within‐generation and transgenerational phenotypic plasticity (Kuijper & Hoyle, 2015). Additionally, other groups have developed systems comparing invasion probabilities of lines with various epigenetic modifier loci (Furrow & Feldman, 2014) and applied information theory (Donaldson‐Matasci, Bergstrom, & Lachman, 2013) to the study of transgenerational phenotypic plasticity.

As formally shown through a variety of models, when environmental autocorrelations increase, the optimal degree of transgenerational response also increases (McNamara, Dall, Hammerstein, & Leimar, 2016). In other words, for transgenerational plasticity to be adaptive, the environment must not only be variable but also predictable (Burgess & Marshall, 2014) from one generation to the next. The scale of environmental variation can also be described in terms of environmental grain (Gillespie, 1974), in which the relative “coarseness” describes whether the environment fluctuates rapidly or slowly between states. When the environmental grain is too coarse, genetic adaptation is expected to predominate over forms of plasticity (Banta, Dole, Cruzan, & Pigliucci, 2007). When the grain is too fine, transgenerational plasticity is not expected to evolve because the environmental information sensed by the parent is out of date when progeny receive it (McNamara et al., 2016). In the case of organisms with relatively fixed generation times, the autocorrelation between parental environmental cues and offspring selective environments provides a simple quantification of the levels of transgenerational plasticity that should maximize the mutual information between phenotype and environment. A common theme across the literature is that these autocorrelations are the most important factor in the adaptive evolution of transgenerational plasticity (Burgess & Marshall, 2014). This consensus motivated us to assess the presence of autocorrelations between successive years, which is the scale of environmental grain that should favor transgenerational plasticity in annual plants or other organisms with similar generation times.

Despite the surge of experimental evidence, molecular understanding, and theoretical interest, no study to date has examined long‐term environmental data across a large area for the presence of such environmental autocorrelations. Although evolutionary research has traditionally focused on how the average environmental conditions differ across a landscape, there is no reason to expect that the scale and predictability of environmental variation are any less complex or ubiquitous than variation in mean environmental conditions. As prior modeling studies have demonstrated (Uller et al., 2015), the *spatial variation* in the *temporal predictability* of environmental variation is expected to drive the evolution of transgenerational effects across a heterogeneous landscape.

In this study, we test whether or not empirical patterns of climatic variation allow for the evolution of within‐generation plasticity, transgenerational plasticity, and multigenerational epigenetic inheritance across different local climate regimes. We use 120 years of fine‐scale (4 km^2^) climate data spanning the coterminous United States to test for auto‐ and cross‐correlations in temperature and precipitation levels across years. We find many significant correlations that vary widely in both magnitude and direction across the United States. We then construct separate models, with summer annual plants in mind, for temperature and precipitation to determine the degree of transgenerational plasticity that would maximize fitness in each of these sites across the United States. Furthermore, by running each model using raw environmental data and the residuals after removing the effects of directional climate change, we are able to inspect how climate change alters the benefits associated with transgenerational plasticity. These results allow us to predict where transgenerational plasticity is expected to evolve given patterns of environmental variation over the past 120 years.

**Figure 1:**
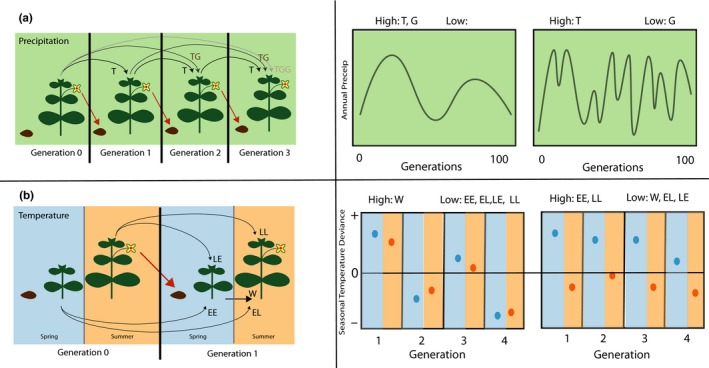
Schematic depicting the ecological motivations (summer annual plants) and theoretical underpinnings for the evolutionary modeling of plasticity traits (a, c), and the types of environmental fluctuations that may influence their evolution (b, d). (a) Precipitation plasticity model. The amount of precipitation experienced by an individual can lead to transgenerational effects in the next generation (T), as well as persist for two (TG) or three (TGG) generations. (b) On the left, relatively gradual decadal oscillations give value to transgenerational effects that persist for multiple seasons (T, TG, and TGG). On the right, shorter period climatic oscillations may favor parental effects (T), but not multigeneration effects (TG or TGG). (c) On the left, we see an example of an environment with high within‐season autocorrelations for temperature (hot springs tend to be followed by hot summers), but low interannual autocorrelations (a hot year does not tend to be followed by another hot year) that select for within‐generation plasticity but not transgenerational plasticity. On the right, a situation where spring and summer temperatures are not correlated with each other, but we do find that environmental oscillations lead to a string of warmer than average springs and cooler than average summers; in this situation, transgenerational plasticity (EE and LL) but not within‐generation plasticity is expected to be optimal. (d) Temperature plasticity model. In the abbreviations, **E** denotes the early growing season (spring), and **L** denotes the late growing season (summer). The first letter represents the relevant season during the parental generation, and the second letter represents the relevant season in the offspring generation (e.g., EL denotes effects of parental early growing season temperature on offspring phenotypes late in the growing season). Within‐generation developmental changes in response to early‐season environment (W) are also considered in this model. Additionally, the long‐term average environmental conditions at a specific area determine the genetic baseline phenotype of an individual (g)

In our precipitation model, we examine transgenerational effects that persist for up to three generations (Figure 1a), as multiple experimental studies have found that environmentally induced epigenetic and phenotypic effects can persist for at least this long (Akkerman, Sattirin, Kelly, & Scoville, 2016; Whittle, Otto, Johnston, & Krochko, 2009), and in some cases for far longer (Rechavi, Minevich, & Hobert, 2011; Vastenhouw et al., 2006). While we do not consider specific mechanisms of transgenerational plasticity, prior work has demonstrated that the offspring of plants exposed to drought stress have higher survival in drought conditions, partially mediated through enhanced root growth phenotypically and altered DNA methylation patterns at the molecular level (Herman & Sultan, 2016). In our temperature model (Figure 1b), we also allow for within‐generation plasticity to early‐season temperature and transgenerational effects impacting both early‐ and late‐season phenotypes. This allows us to compare the predictive quality of information from different seasons as well as the stages in a plant's life cycle that should be most responsive to parental cues. In plants, transgenerational effects of temperature have primarily been demonstrated to shift phenology such as flowering time (Case, Lacey, & Hopkins, 1996) and dormancy (Chen et al., 2014), but other phenotypes such as rosette diameter in Arabidopsis are also impacted by parent temperature (Groot et al., 2017). In animals, egg size, survival, developmental rate, melanization, and heat‐shock survival were all shown to be impacted by parent temperature (*see review*: Donnelson et al. 2018). DNA methylation likely contributes to transgenerational effects of temperature, and small RNAs also appear to be a major contributor (Houri‐Zeevi & Rechavi, 2017).

Our optimality models extend previous models (Kuijper & Hoyle, 2015; Leimar & McNamara, 2015), by considering multiple different seasonal timepoints in the parental and offspring generations in which deterministic transgenerational effects can be induced and alter phenotypes. We also quantitatively simplify prior methods to allow these multiple parameters to be considered across hundreds of thousands of locations. We compare geometric mean fitness across the 120 years of climatic data for individuals that utilize different classes of parental information to different degrees. This approach is similar to how Proulx and Teotonio (2017) used geometric mean fitness to compare invasion success in individuals exhibiting a variety of different maternal effect strategies. Rather than assigning a formal genomic framework to our data, we consider a theoretical scenario in which there is no sexual reproduction, or gene transfer of any kind, and where alleles altering transgenerational plasticity can vary in magnitude and direction. By distilling down our models to identify the optimal values of transgenerational plasticity at a given site, we recapitulate the finding from more dynamic models that plasticity is tied directly to environmental autocorrelations, and we are able to apply these theoretical findings to real‐world climate data. We detect substantial variation in the direction and strength of autocorrelations for temperature and precipitation across the United States; this lends credence to the possibility that inconsistent evidence for transgenerational plasticity between species or populations may be the result of locally adaptive plasticity, rather than methodological discrepancies. Through leveraging this spatial variation in environmental autocorrelations, it should be possible to test hypotheses in nature regarding the ability for transgenerational plasticity to adaptively evolve to match underlying climatic patterns.

## 2 METHODS

### 2.1 Descriptive statistics

Mean monthly temperature and precipitation at a 4‐km resolution from 1895 to 2014 (LT81m) were downloaded from the PRISM Climate Group web server (PRISM Climate Group, ). In short, PRISM uses climate averages from between 1981 and 2010 as a predictor grid and then utilizes station networks with at least 20 years of data to model monthly temperature and precipitation across the United States. The emphasis on this dataset is long‐term consistency making it ideal for our purposes. Individual yearly values were concatenated using the QGIS merge raster function (Quantum GIS geographic information system, 2012) to create a single data frame and exported in the .RData format for downstream analysis. For precipitation data, October was chosen to represent the start of the “hydrologic” year in order to more accurately capture water availability patterns during the growing season. For temperature data, mean daily maximum temperature was calculated for March–May as a measure of early growing season temperature for a given year and July–September for late growing season temperature. Autocorrelations and partial autocorrelations were calculated at lags between 1 and 12 years (i.e., environmental correlations were calculated between year *X* and year *X* + 1, year *X* and year *X* + 2,…,year *X* and year *X* + 12). Partial autocorrelations at lag *X* are the correlations between two timepoints after regressing out the effect of autocorrelations at any shorter timescales.

### 2.2 General modeling framework

Mathematical models were constructed in R for both precipitation and temperature patterns to compare how individuals that use within‐generation plasticity, transgenerational plasticity, and genetic inheritance to varying degrees differ in their capacity to match their phenotype with the environmentally optimal phenotype for a given year. In these models, there are hundreds (precipitation models) or thousands (temperature models) of genotypes, each representing unique points of parameter space for alleles that modify the extent to which environment affects phenotype. Trait value is a measure of the expected environment (temperature or precipitation) and is determined by a combination of the mean environment at a given site over all years and terms that modify this value based on recent environmental information. Each genotype is in essence a climatologist that utilizes genetic information (based on mean precipitation over the 120 years at a site), transgenerational plasticity, and/or within‐generation plasticity (only in temperature model) to come up with an expected environment that it will face. This expected environmental value is equivalent to a phenotype, and the closer the phenotype is to the actual environment experienced, the higher the fitness that genotype will have for a given generation. For both models, two variants were considered, one in which raw data were used as is, and another where linear trends over the 120 years were factored out and residuals were used in the models. By comparing raw and residual models, we gain insight into the relative contribution of directional climate change compared to shorter period oscillations, which may prove useful in identifying regions where transgenerational plasticity proved adaptive even prior to recent warming trends.

For each locale, mean annual precipitation (or temperature) across the 120 years $\left(\bar{P}\right)$ is calculated, and this statistic is used as the baseline phenotype for all genotypes in the raw data variant of the model (Appendix S1). The details of the model are induced in Appendix S1, but they are based on a model in which baseline phenotypes $\left(\bar{P}\right)$ are adjusted by the environment during the prior year $\left(P_{T-1}\right)$ based on a genotypes *m* value ([−1,1] increments of 0.1) such that different genotypes will weigh the contribution of parent environment ($P_{T-1}$) to a different extent. The simplest version of this model has current phenotype $\left({\dot{P}}_{T}\right)$ calculated as ${\dot{P}}_{T}=\bar{P}+m\left(P_{T-1}-\bar{P}\right)$, which is then compared with current environment (*P_T_*) to calculate a phenotype for a given year $w_{\mathrm{MT}}=1-\frac{\left|{\dot{P}}_{T}-P_{T}\right|}{\bar{P}}$ (Appendix S1). When m=0, the expected precipitation will always be equivelant to the mean annual environment at a site, and when m=1, the expected precipitation will always equal the precipitation of the prior year.

While this framework is identical for precipitation and temperature models, inherent differences in precipitation and temperature lead us to expanding these models via the addition of different parameters, allowing us to ask related but unique questions regarding transgenerational inheritance. Precipitation can accumulate as snowpack, bodies of water, or soil moisture, such that the cumulative precipitation over the course of the water year will determine to a large extent the amount of water available to a plant. On the other hand, the effects of temperature are much more immediate and transient, such that a particularly cold spring will not “keep the plant cool” over the summer, in the way that a particularly wet spring could provide moisture during a summer of drought. For this reason, we extend our temperature models to compare patterns across the early (March, April, May: $\bar{P_{E}}$) and late (July, August, September: $\bar{P_{L}}$) growing season, considering plasticity both within a generation (*w*) and transgenerational plasticity with either early‐ or late‐season temperature effecting early‐ or late‐season phenotypes in the following generation (i.e., *m*_EE_ is effect of parent early‐season temperature on offspring early‐season phenotype, Appendix S1). For precipitation, we considered annual hydrologic year precipitation without separating it by seasons, but added the possibility of multigeneration persistence of transgenerational plasticity through the addition of a multigeneration persistence term *g*. Using these extended models, we calculated the phenotypes produced by each of 176 (precipitation) and 3,125 (temperature) genotypes at each site (481,631), for each year (119). Then by comparing the phenotype produced during a season with the actual environment of that season, we imposed a linear cost on fitness based on the distance between phenotype and the actual temperature or precipitation of that year (Appendix S1). Finally, we calculated geometric mean fitness of every genotype at each site independently to predict which genotype would have the greatest increase in frequency over the course of the time series, and we considered this the optimal phenotype for that site.

This modeling framework represents a variant of other transgenerational plasticity models where the direct parent environment alters offspring phenotype. We model the transgenerational effect as a linear reaction norm with slope *m* with respect to the environment experienced at a particular previous point in time. In the case of multigenerational effects, our *g* term (or rather 1‐*g*) linearly reduces the norm of reaction slope of the grandparental and quadratically reduces the norm of reaction slope of the great‐grandparental generation relative to the effects of the parental generation (Appendix S1). This approach is similar to the analytical models designed by Uller et al. (2015) where maternal effects were modeled as a “linear reaction norm with respect to the mother's *perceived* environment” where the perceived environment was the environmental state of the previous generation with an additional normally distributed error term. Leimar and McNamara (2015) utilize a more complex model where adult phenotype is modeled as a logistic function wherein the *a*_mat_ term determines the weighting of maternal environmental cue, as well as two terms (*m*_mat_ and *d*_mat_) that control the weighting of maternal phenotype transgenerational plasticity and direct parent environment transgenerational plasticity. Proulx and Teotonio (2017) consider six different classes of inheritance strategies competing in environments that switch between two states with variable frequencies. In their modeling framework, the strategies aDME and mDME correspond to two‐state variants of positive (*m* > 0) and negative (*m* < 0) transgenerational effects, respectively, as modeled here. Finally, Kuijper and Hoyle (2015) model maternal effects as a linear transgenerational reaction norm but on parental phenotype rather than parental environment. In our models, we consider fitness to decrease linearly as an individual's phenotype moves further from the phenotypic optimum at a point in time. We compare the geometric mean fitness of individuals expressing different strategies over the 120 years to find the strategy most likely to invade. Similar to most previous models (Uller et al., 2015; Proulx & Teotonio, 2017; Lachmann & Jablonka, 1996; but see Leimar & McNamara, 2015; Greenspoon & Spencer, 2018), we base our model on haploid asexually reproducing individuals. Our temperature model extends previous work by explicitly breaking down both the life cycle of the parent and offspring generations between early and late growing season, allowing for five different temporal classes of plasticity (four types of transgenerational plasticity and within‐generation plasticity).

## 3 RESULTS

**Figure 2:**
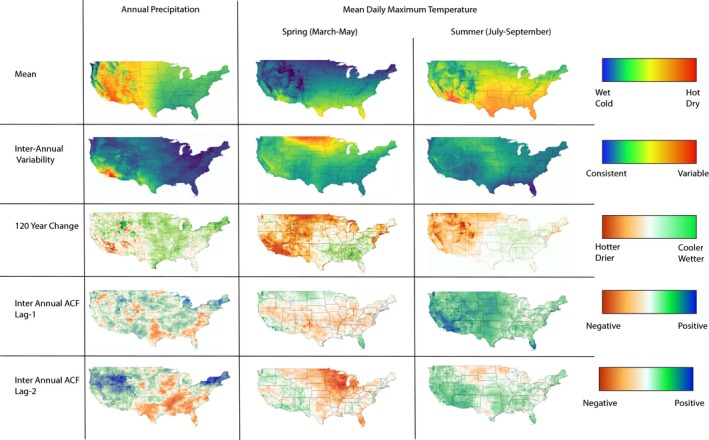
Maps depicting natural climatic variation across the conterminous United States

Both mean annual precipitation and growing season temperatures vary immensely across the United States (Figure 2; Figure S1), but for the evolution of locally adaptive phenotypic plasticity, it is the patterns of variation that are more relevant. The standard deviation of a site's annual precipitation and growing season temperatures over the past 120 years also varied dramatically (Table 1; Table S1), with precipitation interannual standard deviation (IASD) varying from 40 mm to 800 m, spring temperature IASD from 0.77°C to 1.95°C, and summer temperature IASD from 0.35°C to 1.32°C (Figure 2; Figure S1, Table S1). The southwest United States generally had the highest precipitation IASD relative to its mean precipitation, with IASD being nearly equal to the mean precipitation in some regions (Figure 2). Directional climate change over the past 120 years was prevalent and variable across the United States (Figure 2). Mean annual precipitation has declined over much of the Sierra Nevada mountain range, southern California, and other scattered regions over the last 120 years, while precipitation levels have increased in the Midwestern and much of the northeastern United States. Both spring and summer temperatures have risen substantially with the exception of the southeast, where spring temperatures have decreased and summer temperatures have changed little (Figure 2). This phenomenon has been noted numerous times (Ellenburg, McNider, Cruise, & Christy, 2016; Knappenberger, Michaels, & Davis, 2001) and seems to be largely due to a switch from cropland to natural forest ecosystems across the southeastern United States during the past 120 years that has led to greater transpiration cooling.

**Table 1:** Summary statistics of climatic patterns relevant to the evolution of within and transgenerational plasticity

	Mean	IASD	ResACF‐1	ACF‐1	ACF‐2	ACF‐3
Precipitation	763 (443)	145 (76)	0.02 (0.08)	0.04 (0.09)	0.05 (0.13)	0.01 (0.09)
Spring Temp	10.4 (5.5)	1.2 (0.2)	−0.04 (0.07)	−0.01 (0.08)	−0.04 (0.1)	0.05 (0.09)
Summer Temp	21.2 (4.2)	0.9 (0.2)	0.17 (0.1)	0.24 (0.12)	0.09 (0.1)	0.11 (0.09)

Note

Mean (*SD*). IASD, Interannual standard deviation (representative of how variable conditions are between years). ACF, autocorrelation at lags 1, 2, and 3.

Although a variable environment is necessary for the evolution of adaptive phenotypic plasticity, it is the patterns and predictability of this variation that influence which forms of plasticity will be favored. In particular, when the grain of environmental variation is such that autocorrelations between the parental environmental cue and the offspring environment at the time of selection exist, mutual information will be maximized by transgenerational plasticity. We calculated autocorrelations in annual temperature and precipitation levels between successive years, which allowed us to sum across the frequencies of environmental fluctuations to capture the scale of environmental grain expected to favor transgenerational plasticity in annual species. We found that the magnitude and directions of autocorrelations on this timescale were highly variable across the United States (Figure 2, Table 1; Figure S1, Table S1).

Averaged across all sites, the precipitation autocorrelation (AC) at lag‐1 (i.e., the correlation between the precipitation one year and the next) was slightly positive (mean = 0.04, Table 1; Figure S1) and was reduced by half after taking linear changes in precipitation into account (mean = 0.02, Table 1; Figure S1). Spatially, we found that the southeastern gulf coast was the largest region with negative lag‐1 ACF (dry years tend to be followed by wet years), while the northeastern United States was the largest region of substantially positive lag‐1 ACF (Figure 2). Somewhat surprisingly, there were many more sites with moderately positive (62,693: lag‐2 PACF > 0.2, vs. 21,671: lag‐1 ACF > 0.2) and negative (5,088 lag‐2 PACF < −0.2 vs. 441 lag‐1 ACF < −0.2) lag‐2 partial autocorrelation (PACF) than lag‐1 ACF. This suggest that climatic oscillations impacting annual precipitation tend to operate over more than two years in these regions and that on a year‐to‐year basis, variation is more stochastic (leading to lower absolute lag‐1 ACF).

**Figure 3:**
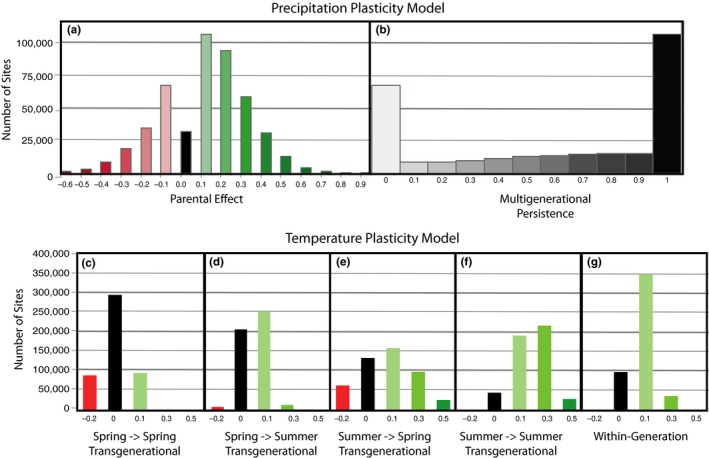
Distributions of optimal plasticity (a, b) and temperature (c–g) values across all 4km x 4km sites in the United States. Histograms of optimal (a) precipitation transgenerational plasticity value (*T*), (b) precipitation multigeneration persistence (*G*), (c) temperature spring (early season) → spring (early season) transgenerational plasticity (*m*_EE_), (d) temperature spring (early season) → summer (late season) transgenerational plasticity (*m*_EL_), (e) temperature summer (late season) → spring (early season) transgenerational plasticity (*m*_LE_), (f) temperature summer (late season) → summer (late season) transgenerational plasticity (*m*_LL_), and within‐generation temperature plasticity (*W*)

Patterns of temperature autocorrelations extended over larger regions and were more extreme than the patterns observed for precipitation autocorrelations (Figure 2). Lag‐1 ACF for spring and summer temperatures varied a great deal, with patterns of summer temperature autocorrelation substantially more positive than those of spring (summer ACF‐1 mean: 0.24; spring mean: −0.01, Figure S1). In both cases, however, the western United States tended to have more positive autocorrelations than the rest of the country (with the exception of southern Florida; Figure 2). The mean lag‐2 PACF for spring temperature was negative (mean: −0.04, Figure 3) and more variable than lag‐1 ACF (*SD* = 0.1 vs. 0.08), with much of the north‐central United States displaying lag‐2 PACF of <−0.2 (Figure 2). The mean lag‐2 PACF for summer temperatures was positive (mean: 0.09), but substantially lower than the mean lag‐1 ACF (mean: 0.24).

Modeling work on transgenerational plasticity has often focused on positive lag‐1 autocorrelations and found them to be highly correlated with optimal transgenerational plasticity. In the particular case of anticipatory transgenerational effects, the autocorrelation between the environment the parent experiences and the offspring selective environment was found to be almost perfectly tied to the evolved mean maternal effects after 50,000 simulated generations (Kuijper et al., 2014), with similar results in a number of other studies (English, Pen, Shea, & Uller, 2015; McNamara et al 2016; Tufto, 2015). Partial autocorrelations at lag‐2 represent the additional mutual information captured by the grandparental environment and are therefore expected to influence the benefits associated with the stability of transgenerational effects over two generations. From these prior modeling results, it is reasonable to expect that locations with high positive autocorrelations may be favorable for the evolution of transgenerational plasticity. Within these sites, areas with high lag‐2 partial autocorrelations may favor the transmission of environmental information across two generations.

## 4 MODELING RESULTS

### 4.1 Optimal levels of transgenerational plasticity: precipitation

As expected, the dramatic variability of precipitation autocorrelations across the United States leads to a great deal of variation in the optimal levels of plasticity in our evolutionary models (Figure 3; Table S1). In the raw variant of the model, optimal parental effect values were positive in 314,118 cases (65%), zero in 32,352 (7%), and negative in 135,161 (28%), compared to 55%, 7%, and 38%, respectively, in the residual variant. The most common “parental effect” value (*m*, see Appendix S1) in the precipitation model was 0.1 (22.5% and 21.8% of sites in the raw and residual models, respectively, Figure 3a.). This level of parental effect indicates that 90% of phenotypic variance is dictated by the long‐term average (genetic effects) and 10% by the difference between the parental environment and the long‐term average environment. The second most common optimal value of *m* was 0.2 (19.15% in the raw model, 18.6% in residual model), followed by −0.1 (14.3% in the raw model, 17.2% in the residual, Figure 3a).

**Figure 4:**
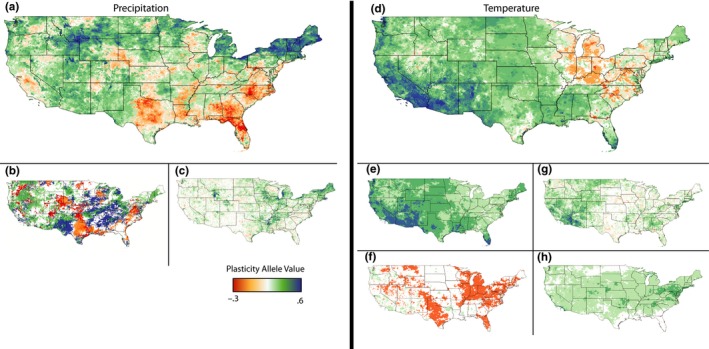
Maps coded to show patterns of variability for optimal precipitation (a–c) and temperature (d–h) plasticity values across the United States. (a) Optimal transgenerational plasticity values for the one‐generation transmission of precipitation level information. (b) Optimal grandparental transgenerational plasticity values coded blue (green) or red (orange) based on the direction of effect (positive or negative). White regions have an optimal multigeneration persistence (*G*) of 0, while red and blue both have optimal multigeneration persistence of 1, intermediate values (0 > *G* > 1) in orange and green. (c) The difference between optimal transgenerational plasticity values in the raw versus. residual variant of the mode. Higher values suggest that the primary value associated with transgenerational plasticity over the past 120 years has been associated with allowing individuals to keep up with linearly changing precipitation patterns. (d) Optimal total levels of transgenerational temperature plasticity ((*M*_EE_ + *M*_EL_ + *M*_LE_ + *M*_LL_)/2). (e) Optimal transgenerational plasticity of most extreme positive transgenerational plasticity allele. (f) Optimal transgenerational plasticity of lowest transgenerational plasticity allele. Regions in orange have at least one form of transgenerational plasticity for which negative transgenerational effects increase fitness. (g) The difference between optimal transgenerational plasticity values in the raw versus residual variant of the mode. Higher values suggest that the primary value associated with transgenerational plasticity over the past 120 years has been associated with allowing individuals to keep up with increasing temperature. (h) Optimal within‐generation plasticity (*W*) values

The multigenerational persistence (*g*, Appendix S1) of transgenerational effects was also found to vary greatly across the United States with the two most common values being 1 (40.7% raw, 39.5% residual) and 0 (18.6% raw, 16.8% residual) (Figure 3b). Here, a value of 1 indicates that the precipitation one, two, and three years prior all contribute equally to the expected precipitation at a given site. A *g* value of 0 indicates that only the previous year's precipitation is predictive of the current precipitation level. The remaining 40.7% of sites (in the raw variant) have intermediate optimal values of *g*, suggesting that in these locations the precipitation of each of the past three years is informative, but information from the immediately preceding year is of the highest value (Figure 4). Interestingly, full multigenerational persistence (*g* = 1) was more frequently optimal at sites with negative transgenerational effect values compared to those with positive values (44.9% vs. 38.9%, respectively), where intermediate multigenerational persistence was more common (Figure S2).

Spatial variation for optimal precipitation plasticity values largely paralleled the spatial distribution of interannual precipitation autocorrelation patterns (compare Figure 2a to Figure 4a). This agrees with previous modeling results that have linked autocorrelation levels with the optimal levels of transgenerational plasticity (McNamara et al., 2016). With grandparental effects included in this model, we find an intriguing portion of parameter space where negative lag‐1 autocorrelations can still favor positive maternal effects (with full multigenerational persistence) if lag‐2 partial autocorrelations are positive (Figure S3, RP). Variation across the landscape for lag‐1 autocorrelations and lag‐2 partial autocorrelations leads to 7 possible strategies of distinct systems of parental effects and multigeneration persistence (Figure S3). This suggests that even in these very simplified models, natural variation in autocorrelations in precipitation can not only favor distinct levels of transgenerational inheritance, but also the persistence of these effects.

At the broadest level, the northern latitudes show the highest optimal transgenerational precipitation plasticity values (Figure 4a), but not necessarily multigeneration persistence of transgenerational effects (Figure 4b). Optimal transgenerational plasticity values were on average 0.057 lower in the residual variant of the model compared to the raw variant, with the vast majority of sites having equal values (56%), decreasing by 0.1 (24%), decreasing by 0.2 (8.5%), or increasing by 0.1 (4.3%). The northeastern United States and the Yellowstone National Park region, where precipitation increased most (Figure 2), also saw the greatest proportion of their optimal transgenerational plasticity values diminished after factoring out linear climate change (Figure 4c). Therefore, although transgenerational plasticity has been optimal over the past 120 years in these regions, these benefits appear to be somewhat contingent upon recent warming trends.

### 4.2 Optimal levels of transgenerational plasticity: temperature

Purely positive transgenerational effects $\left(m_{\mathrm{EE}}\geq 0,m_{\mathrm{EL}}\geq 0,m_{\mathrm{LL}}\geq 0,m_{\mathrm{LE}}\geq 0\right)$ of temperature were optimal in 70.2% of sites (338,327 out of 481,631) in the raw version of the model and 55.7% of sites (268,307) in the residual variant. Conversely, only 1.4% of sites (7,018) in the raw model and 3.1% (14,777) in the residual version included only negative transgenerational plasticity values. Only 0.4% (raw model) or 0.9% (residual model) of sites totally lacked transgenerational plasticity (either positive or negative) as part of the optimal strategy. The optimal strategies in the remaining sites (28% raw model, 40% residual model) comprised a mixture of positive and negative transgenerational plasticity values. Positive within‐generation plasticity was favored in 79.7% of sites (383,667), compared to only 0.03% of sites (157) in which negative within‐generation plasticity (*w*, Appendix S1) was favored, and 20.3% of sites (97,807) in which no within‐generation plasticity was favored (Figure 3g). Optimal levels of within‐generation plasticity were generally positive and minor across the United States; 72% (346,603/481,631) of sites had an optimal *w* value of 0.1 (Table S1).

The most common optimal form of transgenerational plasticity to temperature in both the raw and residual models was the effect of late growing season temperature on the next generation's late growing season phenotype (*m*_LL_, Figure 3f, Table 2a). Effects of late‐season temperature on the next generation's early‐season phenotype (*m*_LE_) were the most variable, with a substantial number of sites having negative transgenerational plasticity values (*m*_LE_ < 0:63,881 raw, 100,267 residual) and many others having moderate (*m*_LE_ = 0.3:98,524 raw, 66,625 residual) and major (*m*_LE_ = 0.5:26,967 raw, 10,080 residual) positive values (Figure 3e). When considering the combined plasticity value profile of a site, the most common combination of plasticity values is *m*_EE_: none (0); *m*_EL_: minor (0.1); *m*_LE_: minor (0.1); *m*_LL_: moderate (0.3); and *w*: minor (0.1) (Table 2b). Summing the four transgenerational plasticity alleles together, we find the southwest United States has the highest optimal values of transgenerational plasticity, while the Great Lakes region has the lowest optimal values (Figure 4d). In the southwestern United States, where temperature increased the most over the past 120 years (Figure 2), the difference between the raw and residual model was the greatest (Figure 4g).

**Table 2:** Most common (#1), second most common (#2), and mean optimal plasticity values across all sites in the United States for precipitation and temperature models

Climate Term	Raw		Residual	
	#1/#2	Mean (*SD*)	#1/#2	Mean (*SD*)
Precipitation				
*m*	0.1/0.2	0.094 (0.22)	0.1/0.2	0.036 (0.21)
*g*	1/0	0.63 (0.40)	1/0	0.64 (0.40)
Temperature				
*m*_EE_	0/0.1	−0.016 (0.098)	0/−0.2	−0.04 (0.11)
*m*_EL_	0.1/0	0.057 (0.073)	0.1/0	0.045 (0.073)
*m*_LE_	0.1/0	0.096 (0.178)	0/0.1	0.042 (0.17)
*m*_LL_	0.3/0.1	0.204 (0.133)	0.1/0.3	0.148 (0.117)
*w*	0.1/0	0.095 (0.072)	0.1/0	0.092 (0.072)

Variation in different classes of temperature autocorrelations between seasons explains a large portion of the variation in the optimal transgenerational response to temperature at a given site. For example, the autocorrelation between early growing season temperature and the next year's late growing season temperature is the factor that explains the largest amount of variation in optimal levels of *m*_EL_ (Table 3). We assessed potential tradeoffs between different forms of transgenerational plasticity to temperature by first calculating the residuals of a particular plasticity term after accounting for the effects of environmental autocorrelations, and then testing the effect of the other four plasticity terms on these residuals. There was a highly significant negative association between *m*_LE_ and *m*_EE_ plasticity, and between *m*_LL_ and *m*_EL_ plasticity (Figure S4a). As higher levels of *m*_LL_ transgenerational plasticity were favored, the optimal levels of EL plasticity also decreased across all environmental autocorrelation values. These associations suggest that, for a given life history stage in this model, there are tradeoffs between using transgenerational information from the previous generation's early versus. late‐season temperature (Table S2). For example, there are many sites where no plasticity, *m*_EE_ plasticity, and *m*_LE_ plasticity all have higher fitness than individuals exhibiting both *m*_EE_ and *m*_LE_ plasticity (Figure S4b).

**Table 3:** Linear regression coefficients of temperature interannual autocorrelations on optimal transgenerational plasticity values

	*m*_EE_	*m*_EL_	*m*_LE_	*m*_LL_
EE ACF	0.757139	−0.131583	−0.049501	−0.156768
EL ACF	−0.036229	0.5968488	−0.069245	−0.153818
LE ACF	0.1256068	−0.036048	1.3390581	−0.067451
LL ACF	0.0135772	−0.017808	−0.120773	0.9757164
Within ACF	−0.067861	−0.160476	0.0595802	0.0701295

### 4.3 Fitness landscapes

**Figure 5:**
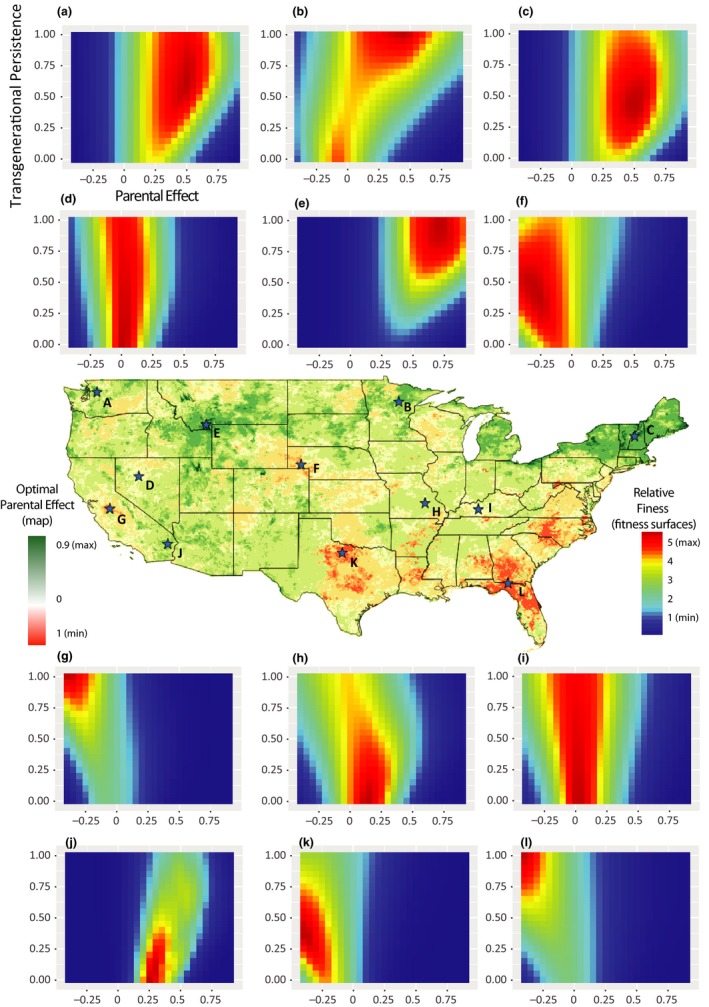
Fitness landscapes of transgenerational precipitation alleles for twelve sites across the United States. Sites with low optimal parental effects (d, i) have only very subtle fitness differences associated with changes in the multigeneration persistence (*y*‐axis) due to the minor role in any form of transgenerational effect on fitness in these cases. More defined fitness peaks tend to occur in areas where more substantialtransgenerational effects are optimal (e, g, j, k, l). In sites with moderately positive (a,c,h) or negative (f) optimal transgeerational plasticity, fitness peaks are less defined as the effects of plasticity alleles are lower. In some cases, bimodal fitness landscapes arise (b) where lines with either positive (with high persistence) or negative (with low persistence) transgenerational persistence have higher fitness than lines with no transgenerational inheritance

In the previous analyses, we used restricted parameter space to identify optimal site‐specific combinations of plasticity values across the entire contiguous United States, but further insight can be gained by comparing fitness landscapes across the full parameter space at individual sites. As both the magnitude of transgenerational plasticity and the persistence of these effects through time have been found to vary, these two parameters represent two biologically realistic components of transgenerational plasticity variation. Using the precipitation model, we found that, among sites where fitness optima are located near zero transgenerational effects, a vertical fitness ridge formed that was centered near parental effect values of zero. This result is due to transgenerational persistence levels (*y*‐axis) having a minimal impact on phenotype when parental effects are marginal. As absolute optimal parental effect values increased, however, the fitness landscape shifted from a ridge to a peak, with certain values of transgenerational persistence imparting extreme fitness advantages over others (Figure 5). Site B (north‐central Minnesota) exemplifies a unique and unexpected outcome of this model: Under certain conditions, there can be multiple local fitness maxima with divergent levels of transgenerational plasticity (Figure 5). Two fitness maxima exist at this site, one in which the optimal strategy comprises slightly negative parental effect values with no multigenerational persistence, and a second in which the optimal strategy comprises slightly to moderately positive parental effect values with high levels of multigenerational persistence. This situation occurs when two conditions hold: The lag‐1 autocorrelation is in a different direction than the average of the lag‐2 and lag‐3 partial autocorrelations, and the absolute value of the lag‐1 autocorrelation is less than the average of the lag‐2 and lag‐3 partial autocorrelations. This scenario occurs in ~90,000 out of the 480,000 sites, but only in 30,000 sites are lag‐2 and lag‐3 average values greater than 0.1 and therefore likely to show up as bimodal peaks in our model. In Figure S3, these points are found in the lower right and upper left quadrants.

## 5 DISCUSSION

Although transgenerational environmental effects on phenotypic expression have been recognized for decades (Falconer, 1981; Roach & Wulff, 1987), interest in these effects has surged recently due to increased appreciation for the potential role of transgenerational plasticity in adaptation (Donelson, Salinas, Munday, & Shama, 2018). Despite this renewed interest, a critical question has remained unanswered: Do natural patterns of environmental variation contain fluctuations of intermediate environmental grain that favor the evolution of adaptive transgenerational plasticity? Our analysis of 120 years of climatic data from the continental United States revealed that such patterns are indeed widespread. Specifically, we analyzed how interannual variation in precipitation and temperature impacts the optimal mode of adaptation for clonally reproducing organisms with a life cycle meant to mimic that of an annual plant. When there are correlations between the parental and offspring environments, neither traditional genetic selection nor within‐generation plasticity take full advantage of the available information inherent in the environment. Instead, under such correlations selection should favor the genetic evolution of mechanisms that transmit plastic responses from one generation to the next. Absent such correlations, the information provided by the parental environment may not be relevant to offspring and indeed may prove to be maladaptive (reviewed by Herman, Spencer, Donohue, & Sultan, 2014).

Our modeling results revealed that the majority of sites in the contiguous United States experience autocorrelations in precipitation and temperature that should favor the evolution of adaptive transgenerational plasticity. As predicted by other models, the predictability of an environmental variable as measured by its autocorrelation is a major factor driving the optimal level of plasticity (English et al., 2015; Groot et al., 2017; Scheiner, 2016; Sultan & Spencer, 2002). Furthermore, we find that the strength and direction of autocorrelations in precipitation and temperature varied substantially across the United States, and consequently, the optimal levels of plasticity were also highly variable. These results provide novel insight into where transgenerational effects are likely to evolve.

The environmental autocorrelation between successive generations reduces the spectra of environmental oscillations, or the grain of environmental variation, to a metric that is highly relevant to transgenerational plasticity. While the precise relationship between the level of autocorrelation and the optimal degree of transgenerational plasticity can vary depending on the precise modeling conditions, autocorrelations between parental environments and offspring selective environments are consistently associated with environments that select for transgenerational plasticity. A recent synthesis of transgenerational plasticity studies highlighted the importance of considering environmental predictability when designing experiments that test for the presence of adaptive transgenerational plasticity (Yin, Zhou, Lin, Li, & Zhang, 2019). Indeed, some experiments that failed to find evidence of adaptive transgenerational plasticity were in systems where models would not expect such effects to evolve. Our results provide a starting point for biologists looking to design experiments on natural variation in transgenerational plasticity.

### 5.1 Precipitation

Local adaptation to variable water regimes has been a major focus of plant evolutionary ecology for many years, and the literature shows that plants have evolved a wide range of physiological, phenological, and morphological adaptations to handle site‐specific patterns of water availability (Kooyers, 2015). These adaptive phenotypes may be expressed constitutively or may be induced by an environmental cue that predicts a change in water availability later in the life of the organism. Increasingly, experimental studies show that the parental soil moisture regime can also adaptively influence the development of progeny (Alsdurf, Anderson, & Siemens, 2015; Alsdurf, Ripley, Matzner, & Siemens, 2013), providing a third route by which plants can fine tune the phenotypes of their offspring to local soil moisture levels. For instance, in Massachusetts genotypes of the annual plant *Polygonum persicaria*, offspring of drought‐stressed parents make more extensive root systems and deploy them faster in response to drought as compared to offspring of well‐watered parents. This drought‐induced change in growth and development can be inherited for at least two generations, resulting in increased survival of grand‐offspring under severe drought stress (Herman et al., 2012; Sultan, Barton, & Wilczek, 2009). Furthermore, these epigenetic effects of drought are genetically variable in *P. persicaria*: Some genotypes strongly increase root length and biomass in response to parental drought, while other genotypes do so only moderately or not at all (Herman & Sultan, 2016).

Our analysis revealed substantial and spatially variable interannual autocorrelations in precipitation, indicating that precipitation levels in one year are often predictive of precipitation levels up to three years later. For example, across the coterminous United States, lag‐1 interannual precipitation autocorrelations varied from moderately negative (−0.27) to strongly positive (0.69), including some values near zero. In turn, the optimal direction and strength of transgenerational effects of precipitation also varied. Positive parental effects, wherein individuals are developmentally predisposed to perform better in environments that match their parents’ environment, were optimal across more than twice as many regions (65% of sites) as negative transgenerational effects (28% of sites), wherein individuals perform better in a different environment than their parents. Relatively strong parental effect values of 0.3 or higher were optimal in nearly 30% of sites. By contrast, complete absence of parental effects was favored in only 7% of sites.

Multigenerational persistence (*g*) values of 0 (18.7% of sites) and 1 (40.7% of sites) were most common, representing strategies in which transgenerational effects lasted only a single generation or persisted fully to the third generation, respectively. The remaining persistence values were somewhat evenly distributed between 0 and 1 and represent strategies in which environmental information gets passed through three generations, but the environment of recent years is weighted more heavily. While lag‐1 autocorrelations were the primary factor involved in dictating the magnitude of optimal parental effects, lag‐2 partial autocorrelations (Figure S3), and to a lesser extent lag‐3 partial autocorrelations, were the primary factors that dictated multigenerational persistence. This result suggests that underlying patterns of environmental variation may play an unexpectedly complex and integral role in dictating the evolution of information transmission systems.

The optimal level of transgenerational effects varied on multiple scales. On the largest scale, we found that the western and northern United States experience conditions that select for the highest levels of transgenerational plasticity (Figure 4a). There was a striking contrast between the northeast, where positive transgenerational plasticity was generally optimal, and the southeast, where negative transgenerational plasticity predominated. On these intermediate‐to‐large spatial scales, it is likely that natural selection could counteract the homogenizing force of gene flow to generate patterns of locally adaptive transgenerational plasticity to precipitation. Experiments designed to compare transgenerational plasticity to precipitation in individuals derived from these north/south or east/west clines would provide novel evidence for climatic patterns shaping the system of inheritance in individuals. There was also considerable variation in optimal levels of transgenerational plasticity on much finer scales. In some cases, levels of transgenerational plasticity were highly divergent between adjacent sites (e.g., in Texas and Minnesota). In these cases, and particularly for outcrossing species, it is less likely that natural selection would be able to counteract gene flow, perhaps limiting the locally adaptive evolution of transgenerational plasticity.

### 5.2 Temperature

Ambient temperature is vitally important to plant function and fitness, as it impacts the rate of physiological reactions, cues developmental transitions, and in extremes can cause stress and mortality. Plants adapt to variable temperature regimes in a host of ways, including the production of heat‐shock proteins and cold‐response factors, and the development of morphologies that mitigate the experience of temperature extremes. Experimental studies have identified adaptive plastic responses to temperature changes, both within and across generations. For example, ambient temperature in *Arabidopsis thaliana* has been shown to influence the expression and splicing of hundreds of genes, leading to changes in histone methylation (Pajoro, Severing, Angenent, & Immink, 2017), and shifts in flowering time (Donohue, 2009) and other phenotypes (Adams, Stewart, Cohu, Muller, & Demmig‐Adams, 2016) in genotype‐specific ways. Additionally, recent work has demonstrated that effects of temperature on *A. thaliana* plants persist for multiple generations (Groot et al., 2017; Suter & Widmer, 2013a, 2013b; Whittle et al., 2009). In order for these responses to adaptively match phenotypes with environments, there must be substantial correlations in temperature within and between growing seasons.

We found significant autocorrelations in temperature, both within and between years. Within a single growing season, temperatures early and late in the growing season tended to be positively correlated across the United States. Furthermore, we found that the temperatures of the late growing season months (July, August, September) were generally positively autocorrelated between successive years. Interannual correlations between the temperatures of the early growing season months (February, March, April) were often much lower. As expected, we find that, at a given site, the strength of the correlation between the average temperature during the season in which information is gathered and the average temperature during the season when selection occurs is highly predictive of both the type and degree of plasticity that will be favored. For example, warmer than average springs were very often followed by hotter than average summers, and this information yielded benefits via within‐generation responses to temperature in many sites. The optimal strategy in more than 99% of sites across the United States contained some form of transgenerational plasticity, suggesting that environmental oscillations provide valuable information that allows transgenerational plasticity to improve the match between phenotypes and temperature regimes.

The most common form of transgenerational plasticity in this model was late growing season temperature impacting the following generation's phenotype late in the growing season, which matches our expectations based on the patterns of temperature autocorrelation. Interestingly, patterns of environmental oscillations lead to favorable strategies in which the current late‐season phenotype was more strongly impacted by the previous late‐season temperature than it was by the current generation early‐season temperature. Indeed, this pattern was found in over half of the regions considered (270k/480k). Although intuition suggests that more recent information is of higher value, this result suggests that parental environments can be more predictive of offspring selective environments than environmental cues from earlier in the offspring generation. This result stems from the cyclic nature of seasonal environments (Auge, Leverett, Edwards, & Donohue, 2017). Since autocorrelations between consecutive early growing seasons were generally low, it is not surprising that effects of early growing season temperatures on phenotypes in the following early growing season were the least common form of plasticity and were in the negative direction more often than the positive.

The west coast of the United States and southern Florida experienced the highest optimal transgenerational plasticity values. Because these regions are due east of large bodies of water, their climates are heavily influenced by maritime airflow including the prevailing westerlies, loop current, and Coriolis effect (Lorentz, 1966). As water has a substantially higher heat capacity than either rock or soil, the location of these land masses downstream of maritime air may predispose them to temperature autocorrelations between years, but whether this result is universal will take studies on other continents. These areas may be primed for large‐scale, community‐level comparisons of transgenerational plasticity. Comparisons of transgenerational plasticity in individuals found on the west versus east coast could shed light on the generality of these patterns across a diversity of annual plants and other taxa.

We found highly variable associations between late growing season temperature and the following generation's early growing season temperature. This result is intriguing because the temperature experienced during seed maturation strongly influences the dormancy and germination behavior of seeds, with cascading effects throughout the life cycles of annual plants (Burghardt, Edwards, & Donohue, 2016; Donohue, 2009). Consequently, site‐specific correlations between maternal late‐season temperature and the early‐season temperature in the next generation may select for divergent, site‐specific effects of maternal temperature on germination. Intriguingly, parental effects of temperature on germination and flowering time are highly genetically variable in *A. thaliana* (Burghardt et al., 2016; Groot et al., 2017; Kerdaffrec & Nordborg, 2017). In *Plantago lanceolata*, such genotype‐by‐maternal temperature effects persist throughout the offspring life cycle to generate variation in reproduction in the field (Lacey & Herr, 2000). Our results suggest that genetic variation for maternal effects may derive in part from variable selection imposed by differences among sites in temperature autocorrelations (see also Groot et al., 2017).

## 6 COMMON THEMES AND FUTURE DIRECTIONS

Although our precipitation and temperature models yielded distinct insights into the dynamics of each of these factors, common themes emerged in both sets of analyses. For example, we found higher levels of interannual autocorrelation, and therefore more prominent transgenerational effects, at northern latitudes and along coastal regions within both models. Studies that compare patterns of transgenerational plasticity across large geographic regions will be necessary to determine whether underlying differences in environmental patterns do in fact drive differences in transgenerational plasticity. While the scale of gene flow varies greatly among species, and migration levels have a complex relationship with the evolution of transgenerational plasticity (Greenspoon & Spencer, 2018), these large‐scale patterns generate large contiguous regions with divergent optimal levels of transgenerational plasticity that should provide ample opportunity for natural selection to drive the evolution of transgenerational effects even in the face of gene flow. For example, in our temperature model, the western half of the United States represents a contiguous region where positive transgenerational effects are expected to evolve, while the neighboring great lakes region is many thousands of square miles in area with negative optimal transgenerational plasticity. These large regional differences should allow selection to produce divergent transgenerational norms of reaction; future studies explicitly modeling the migration and evolutionary parameters of specific species will be necessary to test these predictions in different scenarios.

Another common finding of both the temperature and precipitation models is that transgenerational effects are expected to provide greater benefits in changing climates relative to purely oscillating climates, in which linear climate change has been removed (i.e., the residual models). These results suggest that transgenerational effects may have an important role in adaptation to human‐induced climate change and that rapid climate change should select for more transgenerationally plastic individuals. However, there is an important caveat. In our models, we assume that genotypes are uniform in their mean phenotype, and do not allow for mutations that could lead to genetic adaptation to changing conditions. The potential for transgenerational plasticity to either promote or hinder genetic adaptation has been explored (Day & Bonduriansky, 2011), but our models do not address this issue. In the absence of genetic evolution, it follows that if there is a linear trend toward hotter or drier years in addition to climatic oscillations (as in the raw model variants), then there is more transgenerational information relative to a situation in which only climatic oscillations are occurring (residual model variants). Theory indicates that these dynamics become much more complex when local genetic adaptation to changing conditions is allowed to occur along with plastic responses (Groot et al., 2017). For instance, in some scenarios transgenerational effects can increase fitness in the short term, while reducing it in the long term (Hoyle & Ezard, 2012).

Temperature and precipitation autocorrelations likely stem in part from the same broad‐scale climatic oscillations, such as the El Niño Southern Oscillation (Yang et al., 2018), the Quasi‐biennial oscillation (Baldwin et al., 2001), and the Pacific Decadal Oscillation (Mantua & Hare, 2002; Newman et al., 2016). Aside from these climatic oscillations, autocorrelations will arise due simply to “red” or “pink” noise in which rare, large events and common, small events have equal power in explaining variation (Szendro, Vincze, & Pink‐noise behaviour of biosystems., 2001). It has been demonstrated that even without clear underlying phenomena explaining variation, pink noise is often the model that best explains patterns of ecological and abiotic time‐series variation (Halley, 1996). These oscillations and general patterns of red noise will interact with each other to varying degrees across different regions of the United States, leading to variable levels of autocorrelation at all lags for both precipitation and temperature.

Furthermore, because temperature and precipitation interact to alter moisture availability, it is likely that organisms do not process temperature and precipitation information independently, but rather use them in tandem along with other sources of information to fine tune phenotypes for the most likely future environment. For instance, temperature influences water availability by influencing rates of evaporation and transpiration. Interactions between temperature and water availability also shape the collection of herbivores, pathogens, and competitors present in a given locality. Understanding how these environmental factors jointly influence the expression of transgenerational plasticity is an important goal for future research. A key element of this research direction is to study environmental (auto)correlations at fine scales in the context of dispersal distances. It is possible that transgenerational plasticity may be a more common mode of adaptation for organisms with short dispersal distances, in which parents and offspring are more likely to grow and develop in similar microsites. Finally, differences in life history strategies and generation times will alter the timescales and types of environmental autocorrelations relevant to transgenerational plasticity.

A recent meta‐analysis of 1,170 transgenerational plasticity effect sizes found that there was substantial evidence for adaptive transgenerational plasticity, but that these effects varied according to the type of trait that was considered, the environmental context, and the taxonomic and life history group of the focal organism (Yin et al., 2019). In particular, this meta‐analysis found that annual plants displayed the most substantial evidence for adaptive transgenerational plasticity and that physiological traits showed the highest evidence for adaptive plasticity to parent environments. The finding that annual plants displayed the greatest degree of transgenerational plasticity is consistent with their limited mobility and short life cycle, both of which increase the likelihood that offspring experience similar environments to their parents. The mean effect size found in this study for annual plants was 0.163 for reproductive traits and 0.216 for physiological traits, which is consistent with our modeling results. We found that the mean interannual summer temperature autocorrelation was 0.24 and 0.17 before and after factoring out linear effects of climate change, with optimal transgenerational effect sizes in our temperature model ranging from 0 to 0.3. While Yin et al. (2019) did not consider differences between environmental variables, interannual temperature autocorrelations could drive autocorrelations in a diversity of selective pressures. Taken together with our modeling results, this meta‐analysis indicates that observed strengths of transgenerational effects in annual plants are in line with the predictions made by patterns of autocorrelations observed in nature. Similarly, Yin et al. (2019) found that short‐lived invertebrates were the second most likely group to express transgenerational plasticity, suggesting that the capacity to transmit epigenetic information between generations is not phylogenetically limited. For longer‐lived taxa, interannual autocorrelations would be relatively fine‐grained and thus more likely to select for within‐generation rather than transgenerational plasticity. Future studies modeling the evolution of transgenerational plasticity in individuals with disparate life histories will be critical for better understanding the evolution of these environmental effects.

## 7 CONCLUSION

In summary, we find that patterns of climatic variation in nature favor the adaptive evolution of transgenerational plasticity in organisms with approximately annual generation times, such as annual plants. Our models indicate that variable patterns of climatic autocorrelations  across the United States lead to strikingly different optimal patterns of transgenerational plasticity. Thus, for a given species, one may expect that environmental variation across its range not only selects for different locally adapted mean trait values, but also different classes and magnitudes of plasticity. Perhaps the most meaningful result of this study is that the climatic patterns across the United States vary so dramatically that the optimal value of transgenerational plasticity ranges from extremely high (max ACF=0.75) to nonexistent. It should therefore be expected that although many species, environmental variables, or phenotypes of interest may show no evidence of transgenerational plasticity, such results may be due to their specific ecological situation rather than a fundamental biological limitation. This consideration applies equally strongly to the other side of the coin: because a single population or species expresses strong transgenerational plasticity does not mean that transgenerational effects are a universally vital driver of evolutionary processes. Rather, variation in transgenerational plasticity should be expected, just as genetic variation is ubiquitous in natural populations. Transgenerational plasticity is best considered in the specific ecological and evolutionary context of the study organism, and broad generalizations about the role of these effects in evolution should be avoided until considerably more field data are in hand. The results described here provide a source of testable predictions for geographic variation in this mode of adaptation.

## CONFLICT OF INTEREST

None declared.

## AUTHOR CONTRIBUTIONS

J.C. conceived and performed climate analysis and evolutionary modeling. J.C and J.H. contributed to manuscript preparation and writing.

## Data Availability

All modeling results are publicly available within the supplemental tables and code on GitHub at https://github.com/Methylflower/optimal-environmental-plasticity

## References

[ece36022-bib-0001] Adams, W. W. , Stewart, J. J. , Cohu, C. M. , Muller, O. , & Demmig‐Adams, B. (2016). Habitat temperature and precipitation of *Arabidopsis thaliana* ecotypes determine the response of foliar vasculature, photosynthesis, and transpiration to growth temperature. Frontiers in Plant Science 7, 1026 10.3389/fpls.2016.01026 27504111 PMC4959142

[ece36022-bib-0002] Agrawal, A. , Laforsch, C. , & Tollrian, R. (1999). Transgenerational induction of defences in animals and plants. Nature, 401, 60–63. 10.1038/43425

[ece36022-bib-0003] Akkerman, K. , Sattirin, A. , Kelly, J. K. , & Scoville, A. G. (2016). Transgenerational plasticity is sex‐dependent and persistent in yellow monkeyflower (*Mimulus guttatus*). Environmental Epigenetics, 10.1093/eep/dvw003 PMC5804517 29492285

[ece36022-bib-0004] Alsdurf, J. , Anderson, C. , & Siemens, D. H. (2015). Epigenetics of drought‐induced trans‐generational plasticity: Consequences for range limit development. AoB Plants, 8, plv146 10.1093/aobpla/plv146 26685218 PMC4722181

[ece36022-bib-0005] Alsdurf, J. D. , Ripley, T. J. , Matzner, S. L. , & Siemens, D. H. (2013). Drought‐induced trans‐generational tradeoff between stress tolerance and defence: Consequences for range limits? AoB Plants, 5, plt038 10.1093/aobpla/plt038 24307931 PMC3849778

[ece36022-bib-0006] Auge, G. A. , Leverett, L. D. , Edwards, B. R. , & Donohue, K. (2017). Adjusting phenotypes via within‐ and across‐generational plasticity. New Phytologist, 216, 343–349. 10.1111/nph.14495 28262950

[ece36022-bib-0007] Baldwin, M. P. , Gray, L. J. , Dunkerton, T. J. , Hamilton, K. , Haynes, P. H. , Randel, W. J. , … Takahashi, M. (2001). The quasi‐biennial oscillation. Reviews of Geophysics, 39, 179–229. 10.1029/1999rg000073

[ece36022-bib-0008] Banta, J. A. , Dole, J. , Cruzan, M. B. , & Pigliucci, M. (2007). Evidence of local adaptation to coarse‐grained environmental variation in *Arabidopsis thaliana* . Evolution, 61, 2419–2432. 10.1111/j.1558-5646.2007.00189.x 17711467

[ece36022-bib-0009] Bilichak, A. , Ilnystkyy, Y. , Hollunder, J. , & Kovalchuk, I. (2012). The progeny of *Arabidopsis thaliana* plants exposed to salt exhibit changes in DNA methylation, histone modifications and gene expression. PLoS ONE, 7, e30515 10.1371/journal.pone.0030515 22291972 PMC3264603

[ece36022-bib-0010] Bonduriansky, R. , & Day, T. (2009). Nongenetic inheritance and its evolutionary implications. Annual Review of Ecology, Evolution, and Systematics, 40, 103–125. 10.1146/annurev.ecolsys.39.110707.173441

[ece36022-bib-0011] Burgess, S. C. , & Marshall, D. J. (2014). Adaptive parental effects: The importance of estimating environmental predictability and offspring fitness appropriately. Oikos, 123(7), 769–776. 10.1111/oik.01235

[ece36022-bib-0012] Burghardt, L. T. , Edwards, B. R. , & Donohue, K. (2016). Multiple paths to similar germination behavior in *Arabidopsis thaliana* . New Phytologist, 209, 1301–1312. 10.1111/nph.13685 26452074

[ece36022-bib-0013] Case, A. L. , Lacey, E. P. , & Hopkins, R. G. (1996). Parental effects in *Plantago lanceolata* L. II. Manipulation of grandparental temperature and parental flowering time. Heredity, 76, 287–295.

[ece36022-bib-0014] Chen, M. , MacGregor, D. R. , Dave, A. , Florance, H. , Moore, K. , Paszkiewicz, K. , … Penfield, S. (2014). Maternal temperature history activates Flowering Locus T in fruits to control progeny dormancy according to time of year. Proceedings of the National Academy of Sciences, 111(52), 18787–18792. 10.1073/pnas.1412274111 PMC4284563 25516986

[ece36022-bib-0015] Colicchio, J. (2017). Transgenerational effects alter plant defence and resistance in nature. Journal of Evolutionary Biology, 30, 664–680. 10.1111/jeb.13042 28102915 PMC5382043

[ece36022-bib-0016] Colicchio, J. , Miura, F. , Kelly, J. , Ito, T. , & Hileman, L. (2015). DNA methylation and gene expression in *Mimulus guttatus* . BMC Genomics, 16, 507 10.1186/s12864-015-1668-0 26148779 PMC4492170

[ece36022-bib-0017] Conrath, U. , Beckers, G. J. , Langenbach, C. J. , & Jaskiewicz, M. R. (2015). Priming for enhanced defense. Annual Review of Phytopathology, 53, 97–119. 10.1146/annurev-phyto-080614-120132 26070330

[ece36022-bib-0019] Dantzer, B. , Dantzer, B. , Newman, A. E. M. , Newman, A. E. M. , Boonstra, R. , Boonstra, R. , … McAdam, A. G. (2013). Density triggers maternal hormones that increase adaptive offspring growth in a wild mammal. Science, 340, 1215–1217. 10.1126/science.1235765 23599265

[ece36022-bib-0020] Day, T. , & Bonduriansky, R. (2011). A unified approach to the evolutionary consequences of genetic and non‐genetic inheritance. American Naturalist, 178, E18–E36. 10.1086/660911 21750377

[ece36022-bib-0021] Dey, S. , Proulx, S. R. , & Teotónio, H. (2016). Adaptation to temporally fluctuating environments by the evolution of maternal effects. PLoS Biology, 14(2), e1002388 10.1371/journal.pbio.1002388 26910440 PMC4766184

[ece36022-bib-0022] Donaldson‐Matasci, M. , Bergstrom, C. , & Lachman, M. (2013). When unreliable cues are good enough. American Naturalist, 182, 313–327. 10.1086/671161 23933723

[ece36022-bib-0023] Donnelson, J. M. , Salinas, S. , Munday, P. L. , & Shama, L. N. S. (2018). Transgenerational plasticity and climate change experiments: Where do we go from here? Global Change Biology, 24, 13–34. 10.1111/gcb.13903 29024256

[ece36022-bib-0024] Donohue, K. (2009). Completing the cycle: Maternal effects as the missing link in plant life histories. Philosophical Transactions of the Royal Society B: Biological Sciences, 364, 1059–1074. 10.1098/rstb.2008.0291 PMC2666684 19324611

[ece36022-bib-0025] Ellenburg, W. L. , McNider, R. T. , Cruise, J. F. , & Christy, J. R. (2016). Towards an understanding of the Twentieth‐Century cooling trend in the southeastern United States: Biogeophysical impacts of land‐use change. Earth Interactions, 20, 10.1175/ei-d-15-0038.1

[ece36022-bib-0026] English, S. , Pen, I. , Shea, N. , & Uller, T. (2015). The information value of non‐genetic inheritance in plants and animals. PLoS ONE, 10, e0116996 10.1371/journal.pone.0116996 25603120 PMC4300080

[ece36022-bib-0027] Falconer, D. S. (1981). Introduction to quantitative genetics. London, UK: Longman.

[ece36022-bib-0028] Fisher, R. A. (1930). The genetical theory of natural selection. Oxford, UK: Clarendon.

[ece36022-bib-0029] Furrow, R. , & Feldman, M. (2014). Genetic variation and the evolution of epigenetic regulation. Evolution, 68, 673–683. 10.1111/evo.12225 24588347

[ece36022-bib-0031] Ganguly, D. R. , Crisp, P. A. , Eichten, S. R. , & Pogson, B. J. (2017). The Arabidopsis DNA methylome is stable under transgenerational drought stress. Plant Physiology, 175, 1893–1912. 10.1104/pp.17.00744 28986422 PMC5717726

[ece36022-bib-0032] Ghalambor, C. K. , McKay, J. K. , Carroll, S. P. , & Reznick, D. N. (2007). Adaptive versus non‐adaptive phenotypic plasticity and the potential for contemporary adaptation in new environments. Functional Ecology, 21, 394–407. 10.1111/j.1365-2435.2007.01283.x

[ece36022-bib-0033] Gillespie, J. (1974). The role of environmental grain in the maintenance of genetic variation. The American Naturalist, 108(964), 831–836.

[ece36022-bib-0034] Graham, J. K. , Smith, M. L. , & Simons, A. M. (2014). Experimental evolution of bet hedging under manipulated environmental uncertainty in *Neurospora crassa* . Proceedings of the Royal Society B, 281(1787), 20140706. 24870047 10.1098/rspb.2014.0706 PMC4071552

[ece36022-bib-0035] Greenspoon, P. , & Spencer, H. (2018). The evolution of epigenetically mediated transgenerational plasticity in a subdivided population. Evolution. 10.1111/evo.13619 30298912

[ece36022-bib-0036] Groot, M. P. , Kubisch, A. , Ouborg, N. J. , Pagel, J. , Schmid, K. J. , Verger, P. , & Lampei, C. (2017). Transgenerational effects of mild heat in *Arabidopsis thaliana* show strong genotype specificity that is explained by climate at origin. New Phytologist, 25(3), 1221–1234. 10.1111/nph.14642 28590553

[ece36022-bib-0037] Halley, J. (1996). Ecology, evolution, and 1/f noise. Trends in Ecology & Evolution, 11, 33–37. 10.1016/0169-5347(96)81067-6 21237757

[ece36022-bib-0038] Herman, J. J. , Spencer, H. G. , Donohue, K. , & Sultan, S. E. (2014). How stable 'should' epigenetic modifications be? Insights from adaptive plasticity and bet hedging. Evolution, 68, 632–643. 10.1111/evo.12324 24274594

[ece36022-bib-0039] Herman, J. J. , & Sultan, S. E. (2011). Adaptive transgenerational plasticity in plants: Case studies, mechanisms, and implications for natural populations. Front. Plant Sci., 2, 1–10. 10.3389/fpls.2011.00102 22639624 PMC3355592

[ece36022-bib-0040] Herman, J. J. , & Sultan, S. E. (2016). DNA methylation mediates genetic variation for adaptive transgenerational plasticity. Proceedings of the Royal Society B, 283, 20160988 10.1098/rspb.2016.0988 27629032 PMC5031651

[ece36022-bib-0041] Herman, J. J. , Sultan, S. E. , Horgan‐Kobelski, T. , & Riggs, C. (2012). Adaptive transgenerational plasticity in an annual plant: Grandparental and parental drought stress enhance performance of seedlings in dry soil. Integrative and Comparative Biology, 52, 77–88. 10.1093/icb/ics041 22523124

[ece36022-bib-0042] Holeski, L. M. (2007). Within and between generation phenotypic plasticity in trichome density of *Mimulus guttatus* . Journal of Evolutionary Biology, 20, 2092–2100. 10.1111/j.1420-9101.2007.01434.x 17903186

[ece36022-bib-0043] Holeski, L. M. , Jander, G. , & Agrawal, A. A. (2012). Transgenerational defense induction and epigenetic inheritance in plants. Trends in Ecology & Evolution, 27, 618–626. 10.1016/j.tree.2012.07.011 22940222

[ece36022-bib-0044] Houri‐Zeevi, L. , & Rechavi, O. (2017). A matter of time: Small RNAs regulate the duration of epigenetic inheritance. Trends in Genetics., 33(1), 46–57. 10.1016/j.tig.2016.11.001 27939252

[ece36022-bib-0045] Hoyle, R. B. , & Ezard, T. H. (2012). The benefits of maternal effects in novel and in stable environments. Journal of the Royal Society, Interface, 9, 2403–2413. 10.1098/rsif.2012.0183 PMC3427511 22572028

[ece36022-bib-0046] Jablonka, E. (2013). Epigenetic inheritance and plasticity: The responsive germline. Progress in Biophysics and Molecular Biology, 111, 99–107. 10.1016/j.pbiomolbio.2012.08.014 22975443

[ece36022-bib-0047] Kerdaffrec, E. , & Nordborg, M. (2017). The maternal environment interacts with genetic variation in regulating seed dormancy in Swedish *Arabidopsis thaliana* . PLoS ONE, 12, e0190242 10.1371/journal.pone.0190242 29281703 PMC5744996

[ece36022-bib-0048] Knappenberger, P. C. , Michaels, P. J. , & Davis, R. E. (2001). Nature of observed temperature changes across the United States during the 20th century. Climate Research, 17, 45–53. 10.3354/cr017045

[ece36022-bib-0049] Kooyers, N. J. (2015). The evolution of drought escape and avoidance in natural herbaceous populations. Plant Science, 234, 155–162. 10.1016/j.plantsci.2015.02.012 25804818

[ece36022-bib-0050] Kuijper, B. , & Hoyle, R. B. (2015). When to rely on maternal effects and when on phenotypic plasticity? Evolution, 69, 950–968. 10.1111/evo.12635 25809121 PMC4975690

[ece36022-bib-0051] Kuijper, B. , Johnstone, R. A. , & Townley, S. (2014). The evolution of multivariate maternal effects. PLoS Computational Biology, 10, e1003550 10.1371/journal.pcbi.1003550 24722346 PMC3983079

[ece36022-bib-0052] Lacey, E. , & Herr, D. (2000). Parental effects in *Plantago lanceolata* L. III. Measuring parental temperature effects in the field. Evolution, 54, 1207–1217. 11005289 10.1111/j.0014-3820.2000.tb00555.x

[ece36022-bib-0053] Lachmann, M. , & Jablonka, E. (1996). The inheritance of phenotypes: An adaptation to fluctuating environments. Journal of Theoretical Biology, 181, 1–9. 10.1006/jtbi.1996.0109 8796186

[ece36022-bib-0054] Leimar, O. , & McNamara, J. M. (2015). The evolution of transgenerational integration of information in heterogeneous environments. American Naturalist, 185, E55–E69. 10.1086/679575 25674697

[ece36022-bib-0055] Lopez Sanchez, A. , Stassen, J. H. , Furci, L. , Smith, L. M. , & Ton, J. (2016). The role of DNA (de)methylation in immune responsiveness of Arabidopsis. The Plant Journal, 10.1111/tpj.13252 PMC5132069 27341062

[ece36022-bib-0056] Lorentz, E. (1966) The circulation of the atmosphere. American Scientist, 54, 402–420.

[ece36022-bib-0057] Mantua, N. , & Hare, S. (2002). The Pacific decadal oscillation. Journal of Oceanography, 58, 35–44.

[ece36022-bib-0058] McNamara, J. M. , Dall, S. R. , Hammerstein, P. , & Leimar, O. (2016). Detection vs. selection: Integration of genetic, epigenetic and environmental cues in fluctuating environments. Ecology letters, 19(10), 1267–1276. 27600658 10.1111/ele.12663

[ece36022-bib-0059] Mousseau, T. A. , & Fox, C. W. (1998). Maternal effects as adaptations. New York, NY: Oxford University Press.

[ece36022-bib-0060] Newman, M. , Alexander, M. A. , Ault, T. R. , Cobb, K. M. , Deser, C. , Di Lorenzo, E. , … Smith, C. A. (2016). The Pacific decadal oscillation, revisited. 2016. Journal of Climate, 29, 4399–4427. 10.1175/JCLI-D-15-0508.1

[ece36022-bib-0061] Nicotra, A. B. , Atkin, O. K. , Bonser, S. P. , Davidson, A. M. , Finnegan, E. J. , Mathesius, U. , … van Kleunen, M. (2010). Plant phenotypic plasticity in a changing climate. Trends in Plant Science, 15, 684–692. 10.1016/j.tplants.2010.09.008 20970368

[ece36022-bib-0062] Pajoro, A. , Severing, E. , Angenent, G. C. , & Immink, R. G. H. (2017). Histone H3 lysine 36 methylation affects temperature‐induced alternative splicing and flowering in plants. Genome Biology., 18, 102 10.1186/s13059-017-1235-x 28566089 PMC5452352

[ece36022-bib-0063] PRISM Climate Group . Oregon State University. http://prism.oregonstate.edu

[ece36022-bib-0064] Prizak, R. , Ezard, T. H. , & Hoyle, R. B. (2014). Fitness consequences of maternal and grandmaternal effects. Ecology and Evolution, 4, 3139–3145. 10.1002/ece3.1150 25247070 PMC4161186

[ece36022-bib-0065] Proulx, S. , & Teotonio, H. (2017). What kind of maternal effects can be selected for in fluctuating environments? American Naturalist, 189, E118–E137. 10.1086/691423 28514627

[ece36022-bib-0066] Quantum GIS geographic information system . (2012). Open source geospatial foundation project. New Delhi, India: Free Software Foundation.

[ece36022-bib-0067] Räsänen, K. , & Kruuk, L. (2007). Maternal effects and evolution at ecological timescales. Functional Ecology, 21, 408–421. 10.1111/j.1365-2435.2007.01246.x

[ece36022-bib-0068] Rasmann, S. , De Vos, M. , Casteel, C. L. , Tian, D. , Halitschke, R. , Sun, J. Y. , … Jander, G. (2012). Herbivory in the previous generation primes plants for enhanced insect resistance. Plant Physiology, 158, 854–863. 10.1104/pp.111.187831 22209873 PMC3271773

[ece36022-bib-0069] Rechavi, O. , Minevich, G. , & Hobert, O. (2011). Transgenerational inheritance of an acquired small RNA‐based antiviral response in *C. elegans* . Cell, 147, 1248–1256. 22119442 10.1016/j.cell.2011.10.042 PMC3250924

[ece36022-bib-0070] Roach, D. , & Wulff, R. (1987). Maternal effects in plants. Annual Review of Ecology and Systematics, 18, 209–235.

[ece36022-bib-0071] Scheiner, S. M. (1993). Genetics and evolution of phenotypic plasticity. Annual Review of Ecology and Systematics, 24, 35–68. 10.1146/annurev.es.24.110193.000343

[ece36022-bib-0072] Scheiner, S. (2016). Habitat choice and temporal variation alter the balance between adaptation by genetic differentiation, a jack‐of‐ all‐trades strategy, and phenotypic plasticity. American Naturalist, 187, 633–646. 10.1086/685812 27104995

[ece36022-bib-0073] Schlichting, C. , & Pigliucci, M. (1998). Phenotypic evolution: A reaction norm perspective. Sunderland, MA: Sinauer.

[ece36022-bib-0075] Shea, N. , Pen, I. , & Uller, T. (2011). Three epigenetic information channels and their different roles in evolution. Journal of Evolutionary Biology, 24, 1178–1187. 10.1111/j.1420-9101.2011.02235.x 21504495 PMC3116147

[ece36022-bib-0076] Sikkink, K. L. , Ituarte, C. M. , Reynolds, R. M. , Cresko, W. A. , & Phillips, P. C. (2014). The transgenerational effects of heat stress in the nematode *Caenorhabditis remanei* are negative and rapidly eliminated under direct selection for increased stress resistance in larvae. Genomics, 104(6), 438–446. 10.1016/j.ygeno.2014.09.014 25283346 PMC4268007

[ece36022-bib-0077] Slaughter, A. , Daniel, X. , Flors, V. , Luna, E. , Hohn, B. , & Mauch‐Mani, B. (2012). Descendants of primed Arabidopsis plants exhibit resistance to biotic stress. Plant Physiology, 158, 835–843. 10.1104/pp.111.191593 22209872 PMC3271771

[ece36022-bib-0080] Sultan, S. E. (2015). Organism and environment: ecological development, niche construction, and adaptation. New York, NY: Oxford University Press.

[ece36022-bib-0081] Sultan, S. E. , Barton, K. , & Wilczek, A. M. (2009). Contrasting patterns of transgenerational plasticity in ecologically distinct congeners. Ecology, 90, 1831–1839. 10.1890/08-1064.1 19694132

[ece36022-bib-0082] Sultan, S. E. , & Spencer, H. G. (2002). Metapopulation structure favors plasticity over local adaptation. American Naturalist, 160, 271–283. 10.1086/341015 18707492

[ece36022-bib-0083] Suter, L. , & Widmer, A. (2013a). Environmental heat and salt stress induce transgenerational phenotypic changes in *Arabidopsis thaliana* . PLoS ONE, 8, e60364 10.1371/journal.pone.0060364 23585834 PMC3621951

[ece36022-bib-0084] Suter, L. , & Widmer, A. (2013b). Phenotypic effects of salt and heat stress over three generations in Arabidopsis thaliana. PLoS ONE, 8, e80819 10.1371/journal.pone.0080819 24244719 PMC3828257

[ece36022-bib-0085] Szendro, P. , Vincze, G. , & Szasz, A. (2001). Pink‐noise behaviour of biosystems. Biophysical Journal, 30, 227–231. 10.1007/s002490100143 11508842

[ece36022-bib-0086] Tufto, J. (2015). Genetic evolution, plasticity, and bet‐hedging as adaptive responses to temporally autocorrelated fluctuating selection: A quantitative genetic model. Evolution, 69, 2034–2049. 10.1111/evo.12716 26140293

[ece36022-bib-0087] Uller, T. (2008). Developmental plasticity and the evolution of parental effects. Trends in Ecology & Evolution, 23, 432–438. 10.1016/j.tree.2008.04.005 18586350

[ece36022-bib-0088] Uller, T. , English, S. , & Pen, I. (2015). When is incomplete epigenetic resetting in germ cells favoured by natural selection? Proceedings of the Royal Society B, 282, 20150682 10.1098/rspb.2015.0682 PMC4528548 26136447

[ece36022-bib-0089] Vastenhouw, N. L. , Brunschwig, K. , Okihara, K. L. , Muller, F. , Tijsterman, M. , & Plasterk, R. H. (2006). Gene expression: Long‐term gene silencing by RNAi. Nature, 442, 882 10.1038/442882a 16929289

[ece36022-bib-0090] Verhoeven, K. J. F. , & van Gurp, T. P. (2012). Transgenerational effects of stress exposure on offspring phenotypes in apomictic dandelion. PLoS ONE, 7, e38605 10.1371/journal.pone.0038605 22723869 PMC3377677

[ece36022-bib-0091] Walsh, M. R. , Castoe, T. , Holmes, J. , Packer, M. , Biles, K. , Walsh, M. , … Post, D. M. (2016). Local adaptation in transgenerational responses to predators. Proceedings of the Royal Society B, 283, 20152271 10.1098/rspb.2015.2271 26817775 PMC4795015

[ece36022-bib-0092] Whittle, C. , Otto, S. , Johnston, M. , & Krochko, J. (2009). Adaptive epigenetic memory of ancestral temperature regime in *Arabidopsis thaliana* . Botany‐Botanique, 87, 650–657.

[ece36022-bib-0093] Wibowo, A. , Becker, C. , Marconi, G. , Durr, J. , Price, J. , Hagmann, J. , … Gutierrez‐Marcos, J. (2016). Hyperosmotic stress memory in Arabidopsis is mediated by distinct epigenetically labile sites in the genome and is restricted in the male germline by DNA glycosylase activity. Elife, 5, e13546 10.7554/eLife.13546 27242129 PMC4887212

[ece36022-bib-0094] Yang, S. , Li, Z. , Yu, J. , Hu, X. , Dong, W. , & He, S. (2018). El Ñino Southern oscillation and its impact in the changing climate. National Science Review, 5(6), 840–857. 10.1090/nsr/nwy046

[ece36022-bib-0095] Yin, J. , Zhou, M. , Lin, Z. , Li, Q. , & Zhang, Y. (2019). Transgenerational effects benefit offspring across diverse environments: A meta‐analysis in plants and animals. Ecology Letters, 22(11), 1976–1986. 10.1111/ele.13373 31436014

