# Supplementary figures and images for: Empirical patterns of environmental variation favor adaptive transgenerational plasticity
Source: Ecol Evol. 2020 Jan 29;10(3):1648–65. doi: 10.1002/ece3.6022 (PMC7029079; doi:10.1002/ece3.6022)

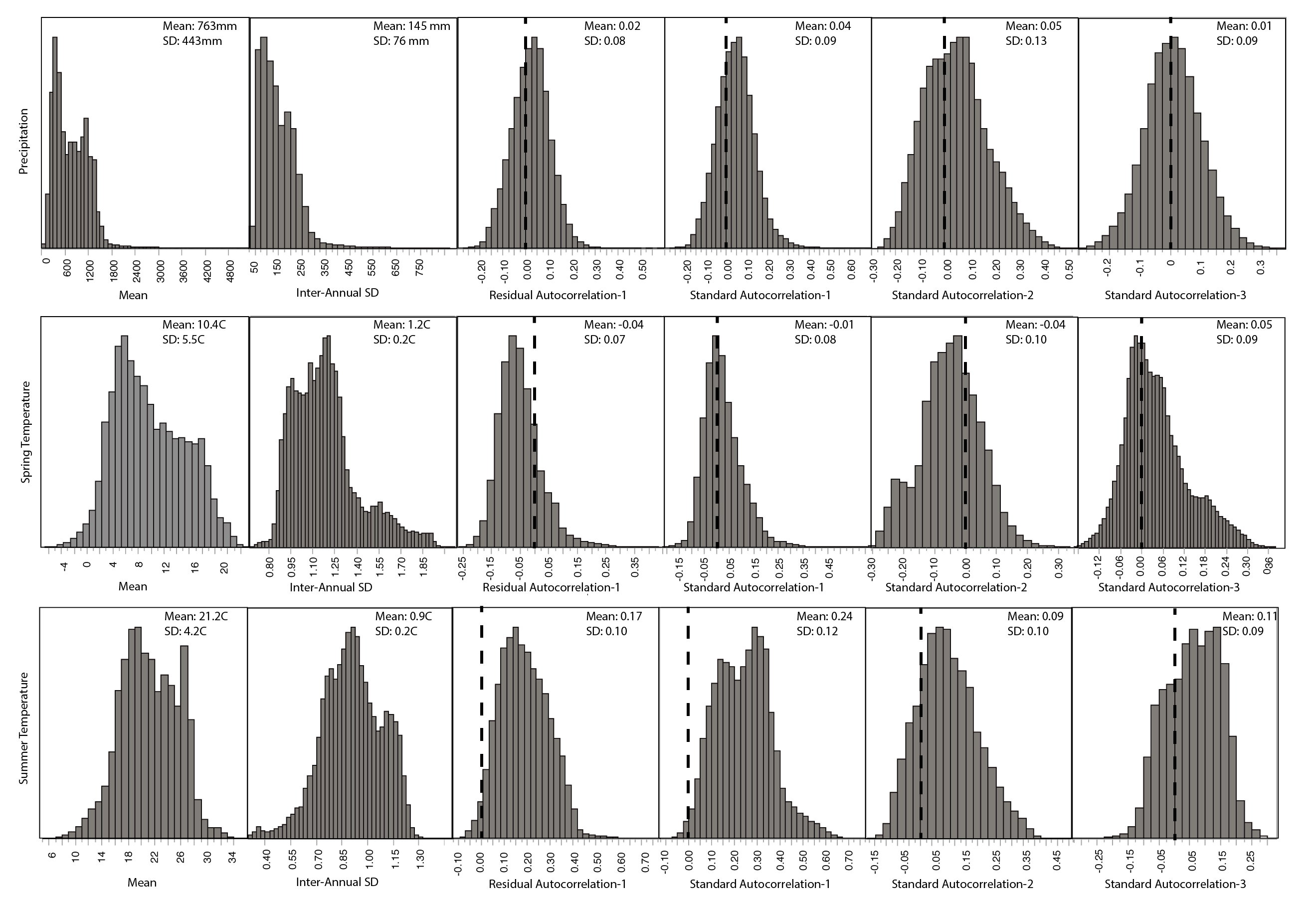

Supplement: Supplementary file 1 [file ECE3-10-1648-s001.png]

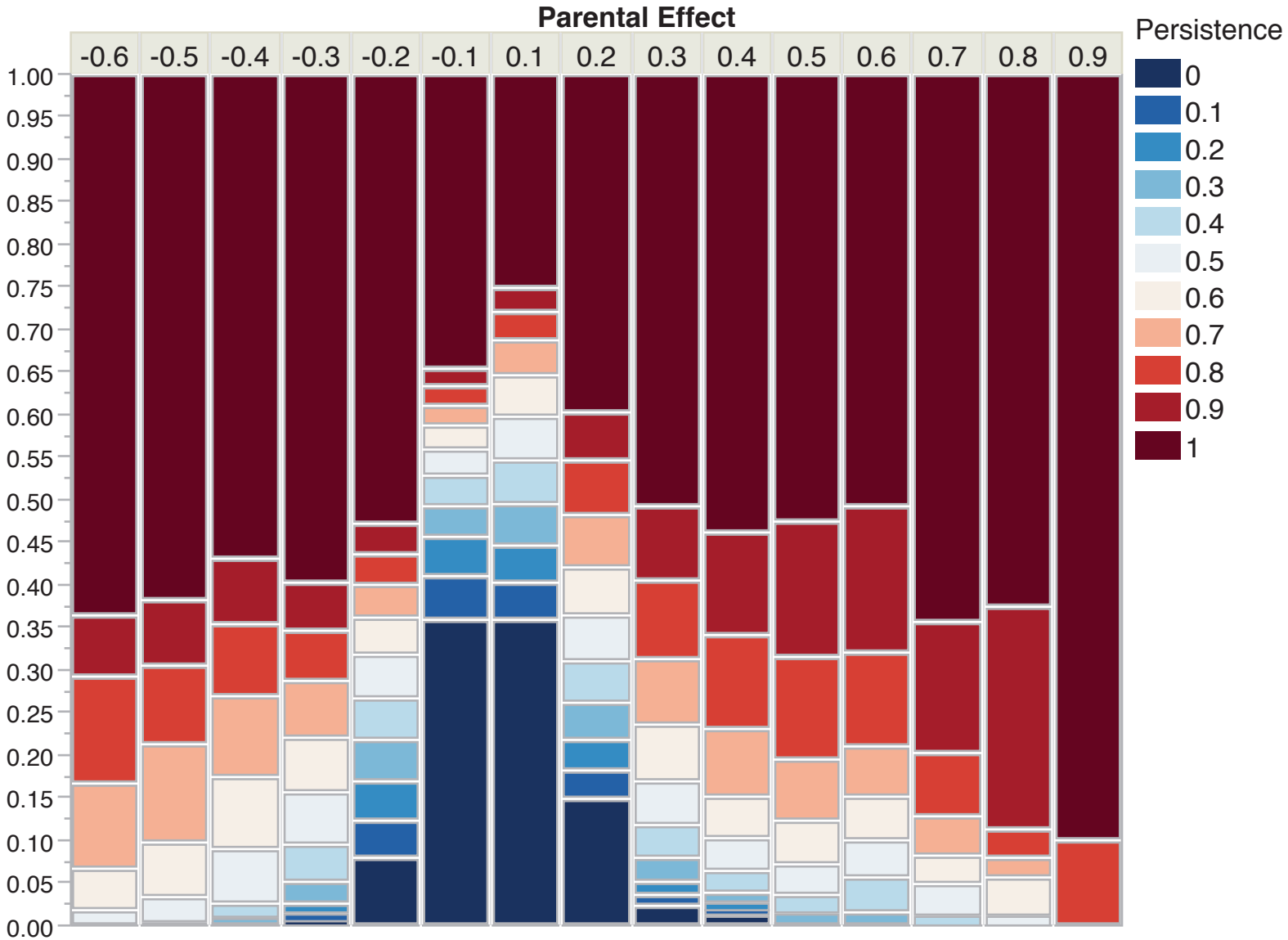

Supplement: Supplementary file 2 [file ECE3-10-1648-s002.pdf]

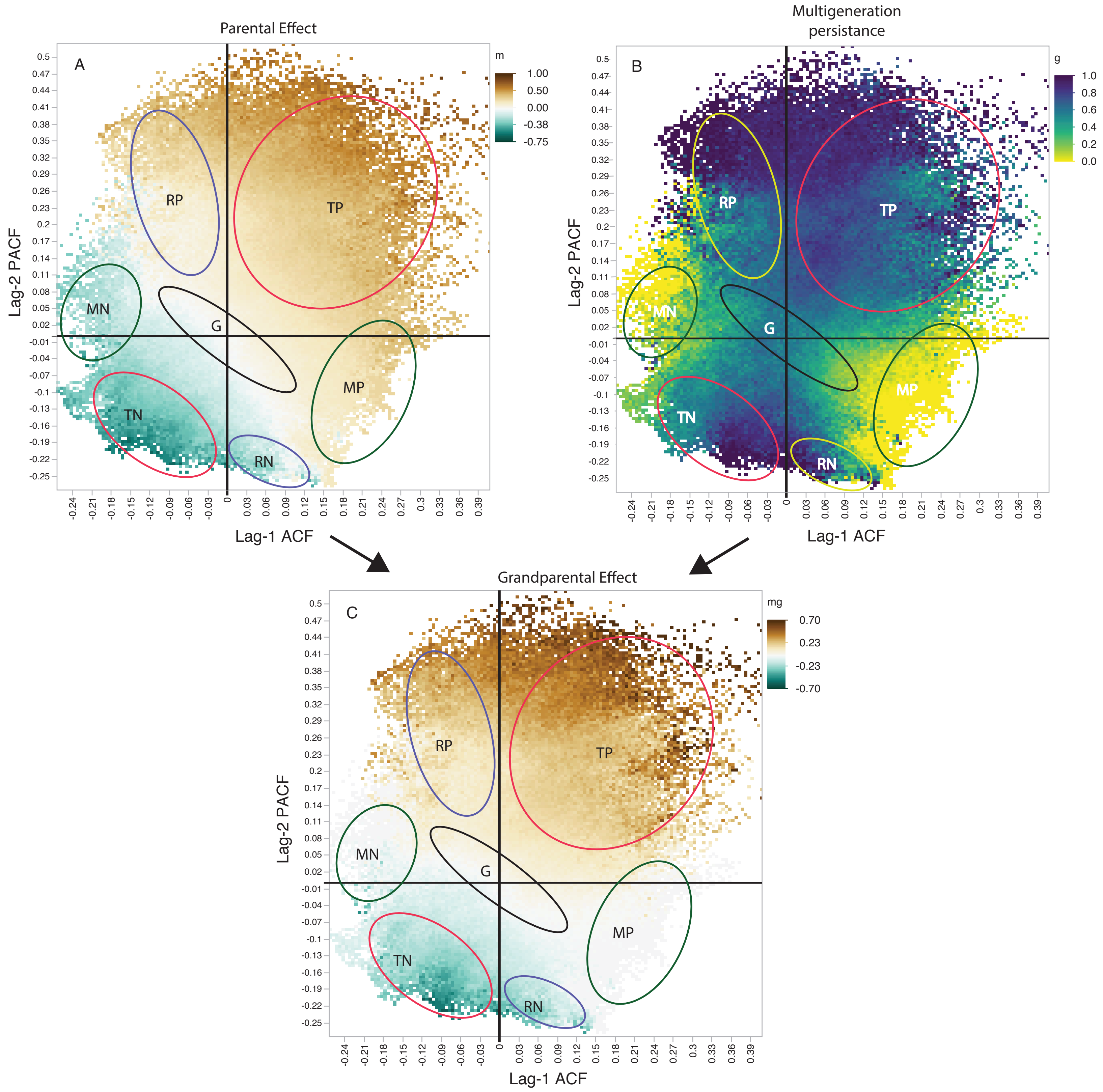

Supplement: Supplementary file 3 [file ECE3-10-1648-s003.pdf]

A

Optimal late-late transgenerational plasticity

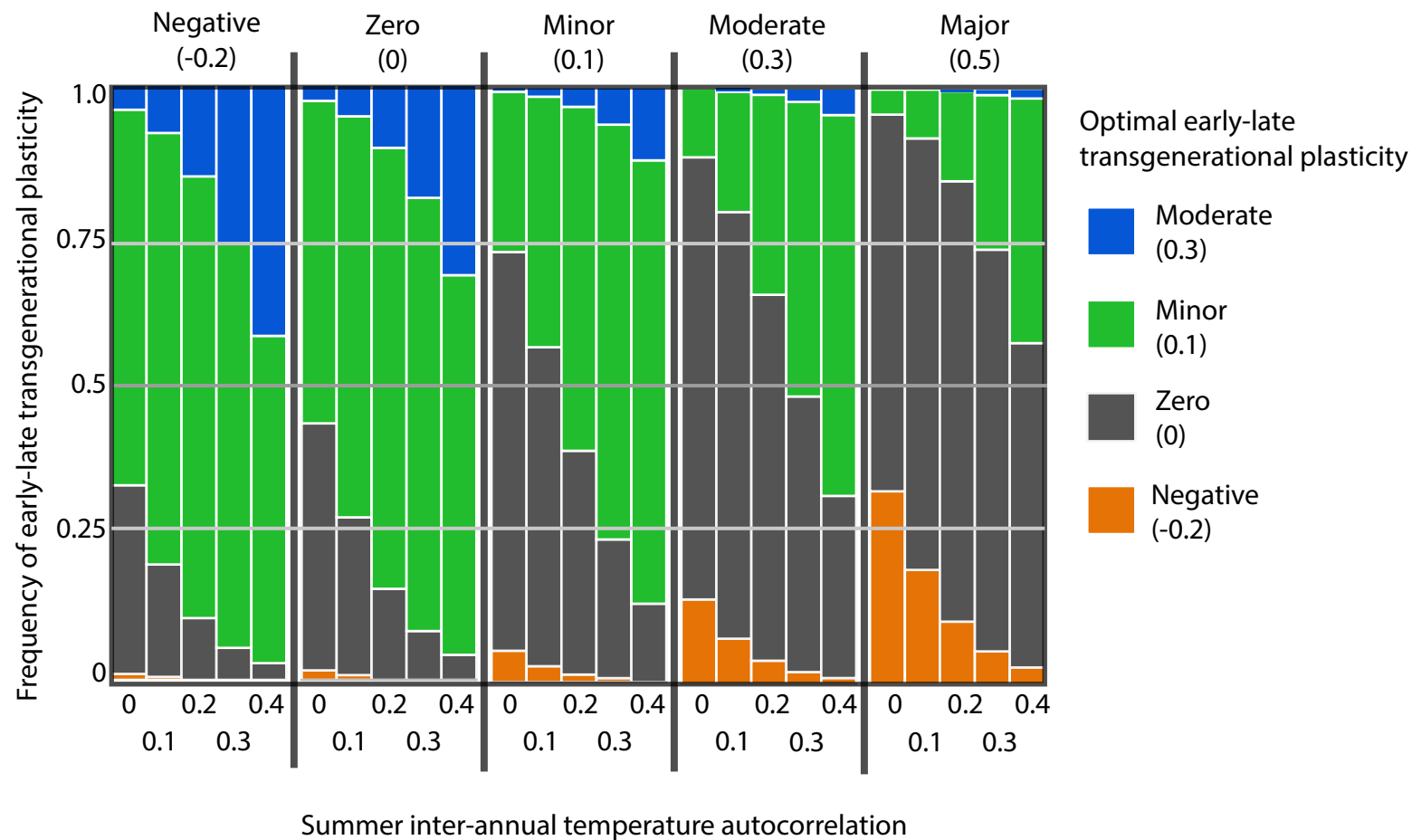

B

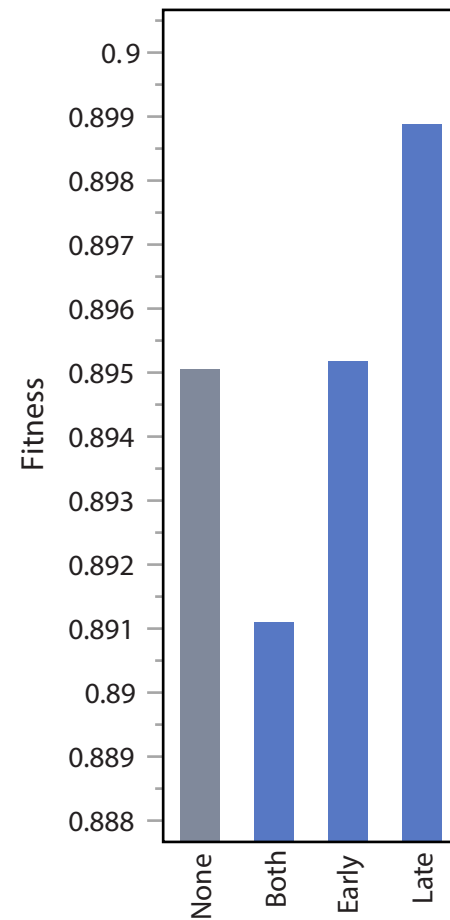

Supplement: Supplementary file 4 [file ECE3-10-1648-s004.pdf]
