# Supplementary material for: Empirical patterns of environmental variation favor adaptive transgenerational plasticity
Source: Ecol Evol. 2020 Jan 29;10(3):1648–65. doi: 10.1002/ece3.6022 (PMC7029079; doi:10.1002/ece3.6022)
Supplement: Supplementary file 7 [file ECE3-10-1648-s007.docx]

Appendix 1

*Precipitation model*

For a given generation *T* the expected precipitation (phenotype) is defined as $\dot{P_{T}}$, while the actual precipitation is $P_{T}$. The two parameters considered in this model are $m$ (maternal or parental effect) and $g$ (multigenerational persistence). The $m$ value in this model alters the expected precipitation (phenotype) for a given year ($\dot{P_{T}}$) based on how deviant the precipitation was during a preceding year or years ${(P}_{T-X}-\bar{P})$. In other words, $m$ adjusts offspring phenotype based on how different parental environment was from long term average environment. When there are interannual autocorrelations, values of m that match the degree of interannual autocorrelation will be adaptive.

$$\dot{P_{T}}=\bar{P}+\frac{m{(P}_{T-1}-\bar{P})+mg\left( P_{T-2}-\bar{P} \right)+mg^{2}(P_{T-3}-\bar{P})}{1+g+g^{2}}$$

When $m=0$ the expected precipitation will always be equivelant to the mean precipitation at a site, when $m=1$ and $g=0$, the expected precipitation will always equal the precipitation experienced the prior year. In the simplest case when $g=0$ only the prior year’s information alters the expected precipitation. The $g$ value in this model weighs how much the precipitation experienced two and three generations back should change expected precipitation values relative to the prior year’s precipitation. At a value of $g=1$ the previous three years will all contribute equally to the expected precipitation. As $g$ decreases, the relative information from two generations back decreases linearly, and the relative information contribution from three generations back decreases quadratically. At $g=0.5$, the grandparental precipitation will contribute half as much information as the parental generation, and the great-grandparental generation will contribute one quarter the information of the parental generation. In other words, the $g$ parameter represents how much the transgenerational information diminishes each year after the first. The cumulative effect of the previous generations environment is scaled by a factor of $(1+g+g^{2})$ in order to standardize the total contribution of transgenerational effects for a given value of $m$ independent of $g$. That is to say, $m$ affects the total amount of transgenerational information transmitted between generations, and $g$ effects the distribution of the information disseminated from each of the prior three years.

A test of a random subset of 2000 sites led us to restrict the values of $m$ we investigated to between -0.6 and 0.9 at increments of 0.1 (from a possible range of -1 to 1). Values less than -0.6 and greater than 0.9 were not optimal in any of the 2000 sites and were therefore excluded for computational reasons. The full range of *g* values (i.e., 0 to 1) was retained. We considered genotypes for every possible combination of $m$ and $g$ values selected at intervals of 0.1 (for a total of 16 × 11 = 176 genotypes). For each genotype we calculated expected phenotype for every year between 1897-2015. Next, for each genotype and each year, we calculated the difference between the expected phenotype and the actual phenotype for that year.

$$w_{MT}=1-\frac{\left| \dot{P_{T}}-P_{T} \right|}{\bar{P}}$$

Geometric mean fitness over the 118 years was calculated for each of the 176 genotypes. The values of $m$ and $g$ for which fitness was maximized was recorded for each of the 481,631 sites and plotted in qGIS. This approach identifies the most fit genotype at a given site over the generations, or more explicitly, the genotype that would be at the highest frequency in the year 2015.

In order to compare evolutionary dynamics in more detail, we constructed fitness landscapes and compared the relative frequency of competing genotypes over time for twelve sites representing a wide range of geographic and parameter space [may need to say more about the parameter space here]. For these sites, the mean fitness across the 176 genotypes was calculated for the first year (1897). The fitness of each genotype in that year was then divided by the mean fitness to produce the relative fitness of each genotype. This relative fitness was then used as the relative frequency of each genotype for the following year. For each year following the first, the absolute fitness of each genotype (*X*) was divided by a weighted mean of the fitness of all individuals for that year ($w_{X}^{Rel}= w_{X}^{Abs}/\sum_{i=1}^{154} w_{i}^{Abs}*Freq(i)$) to calculate the relative fitness of each genotype for each generation. This value was then multiplied by the frequency of that genotype in that generation to get the following generation’s frequency. The final generation frequency was used for the construction of fitness surface heatmaps (Figure 5).

*Temperature model*

The general framework of the temperature model is similar, but rather than allowing for the persistence of transgenerational effects across multiple years, it considers early and late season transgenerational effects, as well as within-generation plasticity. We ran the same model on raw data and residuals, calculated as in the precipitation model. Annual plants were used as the motivation for this model, with a growing season from March-September. The growing season was broken down into three parts: early growing season (March, April, May), transitional growing season (June), and late growing season (July, August, September). Within this model there are two bouts of selection, one after the early growing season, and one after the late season. While early season phenotype can only be modified by the previous year’s temperature (because organisms are newly born in the spring in this scenario, and therefore cannot respond themselves to temperatures), late growing season phenotype can be modified by the previous year’s temperature, and/or the temperature earlier in the growing season. Additionally, this model allows for both early and late season phenotype to be modified by both the previous season’s early and late temperatures independently. In other words, early season phenotype can be modified by the previous year’s early season temperature, or the previous year’s late season temperature; late season phenotype can be modified by these same two factors as well as the current year’s early season temperature (i.e., by within-generation plasticity). Thus, this model includes five plasticity parameters, reflecting the effect of 1) the previous generation’s early season temperature on current early season phenotype ($m_{EE}$), 2) the previous generation’s late season temperature on current early season phenotype ($m_{LE}$), 3) the previous generation’s early season temperature on the current late season phenotype($m_{EL}$), 4) the previous generation’s late season temperature on current late season phenotype ($m_{LL}$), and 5) the current early season temperature on the current late season phenotype ($b$). Note that the mean and variance of temperature data are not correlated, unlike precipitation data. For this reason, fitness is scaled by a constant 20 rather than the mean temperature of a given site. Results were exceedingly similar when fitness was scaled by constants of 10 and 100; 20 was chosen as it is closer to the mean annual temperature across the US.

$$\dot{P_{TE}}=\bar{P_{E}}+ m_{EE}\left( P_{ET-1}-\bar{P_{E}} \right)+ m_{LE}\left( P_{LT-1}-\bar{P_{L}} \right)$$

$$w_{E}=1-\frac{\left| \dot{P_{TE}}-P_{TE} \right|}{20}$$

$$\dot{P_{TL}}=\bar{P_{L}}+ m_{EL}\left( P_{ET-1}-\bar{P_{E}} \right)+ m_{LL}\left( P_{LT-1}-\bar{P_{L}} \right)+ b\left( P_{ET}-\bar{P_{E}} \right)$$

$$w_{L}=1-\frac{\left| \dot{P_{TL}}-P_{TL} \right|}{20}$$

$$w_{F}= w_{E}*w_{L}$$

The geometric mean of genotype fitness was taken over the 119 years for each of the genotypes. For each of the five plasticity (transgenerational and within generational) terms in this model we considered possible values of -0.2 (negative plasticity), 0 (no plasticity), 0.1 (minor plasticity), 0.3 (moderate plasticity), and 0.5 (major plasticity). While this resolution is somewhat coarse, focusing on this subset of values allowed us to consider this model for the full factorial combination of parameter space. In total, fitness was calculated for 3125 genotypes, across each of 119 years, at all 481631 locations, for a total of 179 billion measures of fitness. The values of plasticity at which fitness was maximized were recorded from each grid point across the US. Finally, for the same 12 focal sites used in the precipitation model, we calculated the changes in genotype frequency across 119 years using the same methodology as above. All code for these simulations, and other modeling results are available at (https://github.com/Methylflower/optimal-environmental-plasticity).
